# Flv3A facilitates O_2_ photoreduction and affects H_2_ photoproduction independently of Flv1A in diazotrophic *Anabaena* filaments

**DOI:** 10.1111/nph.18506

**Published:** 2022-10-11

**Authors:** Anita Santana‐Sánchez, Lauri Nikkanen, Elisa Werner, Gábor Tóth, Maria Ermakova, Sergey Kosourov, Julia Walter, Meilin He, Eva‐Mari Aro, Yagut Allahverdiyeva

**Affiliations:** ^1^ Molecular Plant Biology, Department of Life Technologies University of Turku Turku FI‐20014 Finland

**Keywords:** auxiliary electron transport, flavodiiron proteins, H_2_ photo production, heterocyst, Mehler reaction, N_2_‐fixing cyanobacteria, photosynthesis

## Abstract

- The model heterocyst‐forming filamentous cyanobacterium *Anabaena* sp. PCC 7120 (*Anabaena*) is a typical example of a multicellular organism capable of simultaneously performing oxygenic photosynthesis in vegetative cells and O_2_‐sensitive N_2_‐fixation inside heterocysts. The flavodiiron proteins have been shown to participate in photoprotection of photosynthesis by driving excess electrons to O_2_ (a Mehler‐like reaction).
- Here, we performed a phenotypic and biophysical characterization of *Anabaena* mutants impaired in vegetative‐specific Flv1A and Flv3A in order to address their physiological relevance in the bioenergetic processes occurring in diazotrophic *Anabaena* under variable CO_2_ conditions.
- We demonstrate that both Flv1A and Flv3A are required for proper induction of the Mehler‐like reaction upon a sudden increase in light intensity, which is likely important for the activation of carbon‐concentrating mechanisms and CO_2_ fixation. Under ambient CO_2_ diazotrophic conditions, Flv3A is responsible for moderate O_2_ photoreduction, independently of Flv1A, but only in the presence of Flv2 and Flv4. Strikingly, the lack of Flv3A resulted in strong downregulation of the heterocyst‐specific uptake hydrogenase, which led to enhanced H_2_ photoproduction under both oxic and micro‐oxic conditions.
- These results reveal a novel regulatory network between the Mehler‐like reaction and the diazotrophic metabolism, which is of great interest for future biotechnological applications.

## Introduction

Filamentous heterocyst‐forming cyanobacteria such as *Anabaena* sp. PCC 7120 (hereafter *Anabaena*) represent a unique group of prokaryotes capable of simultaneously performing two conflicting metabolic processes: O_2_‐producing photosynthesis in vegetative cells, and O_2_‐sensitive N_2_ fixation in heterocysts (lacking active oxygen‐evolving Photosystem II (PSII)). This ability has evolved through cellular differentiation under nitrogen‐limiting conditions when some vegetative (photosynthetic) cells from the filament transform into specialized heterocyst cells that provide a microaerobic environment suitable for nitrogen (N_2_) fixation. Hydrogen (H_2_) gas is naturally produced as an obligatory by‐product of the N_2_‐fixation process carried out by nitrogenase, which is highly sensitive to oxygen (O_2_). The natural yield of H_2_ inside heterocysts is limited. This is due to rapid H_2_ recycling, mainly by an uptake hydrogenase enzyme, which returns electrons for the N_2_‐fixing metabolism (Bothe *et al*., 2010; Kosourov *et al*., 2014).

In oxygenic photosynthesis, light excites the specific Chl pairs P680 and P700 at the reactions centres of PSII and Photosystem I (PSI), respectively, allowing oxidation of water and subsequent electron transport to NADP^+^, via PSII, Cytochrome (Cyt) *b*_6_ *f* and PSI complexes in the thylakoid membrane, the soluble electron carrier proteins plastocyanin (PC) and ferredoxin (Fd), as well as ferredoxin:NADP^+^‐oxidoreductase (FNR). These electron transport reactions are coupled to ATP synthesis via the generation of a trans‐thylakoid proton motive force (pmf). The NADPH and ATP obtained are then used as reducing power for CO_2_ fixation and cell metabolism. Environmental fluctuations in light and nutrient supply might result in the over‐reduction of the photosynthetic machinery. Alleviation of excessive reduction by class‐C flavodiiron proteins (hereafter FDP) has been described in all oxygenic photosynthetic organisms, apart from angiosperms, and red and brown algae (Helman *et al*., 2003; Zhang *et al*., 2009; Gerotto *et al*., 2016; Chaux *et al*., 2017; Ilík *et al*., 2017; Jokel *et al*., 2018; Alboresi *et al*., 2019; Shimakawa *et al*., 2021). These proteins act as strong electron outlets downstream of PSI by catalysing the photoreduction of O_2_ into H_2_O (the Mehler‐like reaction) (Helman *et al*., 2003; Allahverdiyeva *et al*., 2013, 2015; Santana‐Sánchez *et al*., 2019).

Six genes encoding FDPs have been reported in *Anabaena* (Zhang *et al*., 2009; Ermakova *et al*., 2013). Four of these genes (*flv1A*, *flv3A*, *flv2*, and *flv4*) are highly similar to their homologs in *Synechocystis* sp. PCC 6803 (hereafter *Synechocystis*), SynFlv1–SynFlv4. Recently, we demonstrated that SynFlv1 and SynFlv3 function in coordination with, but distinctly from, SynFlv2 and SynFlv4 (Santana‐Sánchez *et al*., 2019). While the SynFlv1/Flv3 hetero‐oligomer is mainly responsible for the initial fast and transient O_2_ photoreduction during a sudden increase in light intensity (Santana‐Sánchez *et al*., 2019), with Fd acting as the electron donor (Nikkanen *et al*., 2020; Sétif *et al*., 2020), SynFlv2/Flv4 catalyzes steady O_2_ photoreduction under illumination at air‐level CO_2_ (LC). Importantly, a single deletion of any SynFDP strongly diminishes O_2_‐photoreduction, indicating that O_2_ photoreduction is mainly catalyzed by the hetero‐oligomeric forms working in an interdependent manner (Santana‐Sánchez *et al*., 2019; Nikkanen *et al*., 2020).

The two additional *Anabaena* FDP proteins, AnaFlv1B and AnaFlv3B, are exclusively localized in the heterocysts (Ermakova *et al*., 2013). The AnaFlv3B protein was shown to mediate photoreduction of O_2_ independently of AnaFlv1B, likely as a homo‐oligomer, playing an important role in maintaining micro‐oxic conditions inside heterocysts under illumination, which is crucial for N_2_ fixation and H_2_ production (Ermakova *et al*., 2014). However, research into the role of heterocyst‐specific AnaFlv1B and vegetative cell‐specific FDPs in diazotrophic cyanobacteria remains scarce.

Here, we address the physiological relevance of the AnaFlv1A and AnaFlv3A isoforms in the bioenergetic processes in vegetative cells and heterocysts of diazotrophic *Anabaena*. AnaFlv1A and AnaFlv3A are shown to have a crucial role under fluctuating light intensities, regardless of nitrogen or CO_2_ availability, suggesting a functional analogy with homologs in *Synechocystis*. Importantly, our results provide evidence for distinct functional roles of AnaFlv3A and AnaFlv1A. We showed that by cooperating with AnaFlv2 and/or AnaFlv4, AnaFlv3A can function independently of AnaFlv1A in O_2_ photoreduction under low CO_2_ conditions. AnaFlv3A was also indirectly linked with H_2_ metabolism in heterocyst cells. Our work highlights the complex regulatory network between oxygenic photosynthesis, nitrogen fixation and H_2_ photoproduction.

## Materials and Methods

### Strains and culture conditions

*Anabaena* sp. PCC 7120 was used as the wild‐type (WT). The ∆*flv1A* and ∆*flv3A* lines (Allahverdiyeva *et al*., 2013) and the ∆*hupL* mutant (Masukawa *et al*., 2002) have been described previously. The second clone of ∆*flv3A* was constructed by interrupting the *all3895* gene with a neomycin cassette as previously described (Allahverdiyeva *et al*., 2013). For construction of the double mutant Δ*flv1A/flv3A*, the BamHI‐XbaI region of the mutated *flv1A* construct was replaced with a spectinomycin/streptomycin resistance cassette. The plasmid generated was transferred into Δ*flv3A*, followed by selection with sucrose, neomycin, and spectinomycin as described by Cai & Wolk (1990). Segregation of the mutant was verified by polymerase chain reaction (PCR) (Fig. S1d,e). Culture stocks of ∆*flv1A* and ∆*flv3A* were maintained in BG‐11 medium supplemented with 40 μg ml^−1^ neomycin, while Δ*flv1A/flv3A* was additionally supplemented with 25 μg ml^−1^ spectinomycin, and ∆*hupL* was supplemented with 20 μg ml^−1^ spectinomycin.

Pre‐cultures were grown in Z8x medium (lacking combined nitrogen, thus creating N_2_‐fixing conditions, pH 7.0–7.3, Kotai, 1972) at 30°C under constant white light (50 μmol photons m^−2^ s^−1^) without antibiotics. Filaments were inoculated at OD_750_ = 0.1 in 200 ml Z8x medium with continuous bubbling with air (0.04% CO_2_; LC conditions) or air supplemented with 1% CO_2_ (HC conditions) unless mentioned otherwise. Pre‐cultures were harvested at the logarithmic growth phase and inoculated at OD_750_ = 0.1 in fresh Z8x. Experimental cultures were grown similarly to pre‐experimental conditions and harvested after 4 d.

### Determination of heterocyst frequency

Alcian Blue was used to stain the polysaccharide layer of the heterocyst envelope (McKinney, 1953). Cell suspensions were mixed (1 : 8) with a solution of 0.5% Alcian Blue stain in 50% ethanol–water. Stained samples were visualized using a Wetzlar light microscope (Leica, Wetzlar, Germany) and ×400 magnification micrographs were taken. For each sample, 1000–2000 cells were counted, and heterocyst frequency was determined as a percentage of total cells counted.

### Membrane inlet mass spectrometry measurements

*In vivo* fluxes of ^16^O_2_ (*m*/*z* = 32), ^18^O_2_ (*m*/*z* = 36), CO_2_ (*m*/*z* = 44) and H_2_ (*m*/*z* = 2) were monitored using membrane inlet mass spectrometry (MIMS) as described previously (Mustila *et al*., 2016). Harvested filaments were resuspended with fresh Z8x, adjusted to 10 μg ml^−1^ Chl*a* and acclimated for 1 h under growth conditions. For LC samples, dissolved inorganic carbon was saturated with 1.5 mM NaHCO_3_ before measurements were made. Carbon‐concentrating mechanism (CCM) coefficients were determined according to Douchi *et al*. (2019).

To measure the deuterium (D_2_) uptake, filaments were flushed with argon (Ar) for 15 min. Pure D_2_ was injected to reach 2% in the headspace. Changes in H_2_ (*m*/*z* = 2), D_2_ (*m*/*z =* 4) and HD (*m*/*z* = 3) content in the gas phase were measured at 2 and 24 h after the addition of D_2_. For this purpose, 250 μl gas samples from the headspace were injected into the gas‐tight MIMS chamber. Changes in H_2_, D_2_ and HD content were estimated as the difference between the background level and the maximum response on sample injection (Fig. S2). Total H_2_ recycling capacity was estimated as the consumption of D_2_ by the suspension from the headspace, which depends on the total hydrogenase (Hup + Hox) activity in cells. A significant contribution of nitrogenase to D_2_ uptake was excluded since the H/D exchange reaction requires N_2_ (Vignais, 2005). Indeed, in all samples HD was formed at very low concentrations compared to the H_2_ and D_2_ signals (Fig. S2). Therefore, only the change in D_2_ was finally analysed. Calibration of D_2_ concentration was performed by injecting known concentrations of D_2_ into the chamber.

### Chl*a* fluorescence analysis

A Dual‐PAM‐100 spectrophotometer (Walz, Effeltrich, Germany) was used to monitor Chl*a* fluorescence. Harvested filaments were resuspended in fresh Z8x medium to 15 μg ml^−1^ Chl*a*, kept for 1 h under growth conditions, and dark‐adapted for 10 min. A saturating pulse (SP, 5000 μmol photons m^−2^ s^−1^, 400 ms) was administered in darkness to determine *F*_m_^D^, followed by illumination with red actinic light at 50 μmol photons m^−2^ s^−1^ for 380 s while saturating pulses were given every minute (SP1–SP9). Photosynthetic parameters were determined as described previously (Huokko *et al*., 2017).

### Determination of Photosystem I primary donor (P700) and ferredoxin redox changes from near‐infrared absorbance

The absorbance differences at 780–820, 820–870, 840–965 and 870–965 nm were measured with a Dual KLAS/NIR spectrophotometer (Walz). Experimental cultures, and cultures used for the determination of model spectra (Methods S1), were grown at 50 μmol photons m^−2^ s^−1^ under LC conditions in Z8x medium for 4 d, adjusted to 20 μg ml^−1^ Chl*a*, and dark‐adapted for 10 min, after which absorbance differences of the four wavelength pairs were measured during 5 s actinic illumination at 500 μmol photons m^−2^ s^−1^ followed by darkness. The maximal levels of P700 oxidation and Fd reduction were determined for each sample using the NIRMAX script (Klughammer & Schreiber, 2016), and the deconvoluted traces were then normalized to the maximal values. Measurements of *Synechocystis* ∆*flv1* cells were performed as described previously (Nikkanen *et al*., 2020).

### Measurement of the electrochromic shift

The electrochromic shift (ECS) signal was measured as the absorbance difference between 500 and 480 nm (Viola *et al*., 2019) using a JTS‐10 spectrophotometer (BioLogic, Seyssinet‐Pariset, France). Filaments grown under LC conditions were harvested and re‐suspended in fresh Z8x medium at 7.5 μg ml^−1^ Chl*a*, and dark‐adapted for 5 min. Filaments were illuminated at 500 μmol photons m^−2^ s^−1^ for 2 min, with 600 ms dark intervals at specific time points (Fig. S3). Dark interval relaxation kinetics (DIRK) of the ECS signal were monitored to determine the magnitude of the pmf as the light‐induced change in the ECS signal, the conductivity of the thylakoid membrane (*g*H+) as the inverse of the time constant of a first‐order fit to DIRK kinetics, and the thylakoid proton flux (*v*H+) as the product of pmf and *g*H+.

### Hydrogen measurement with a Clark‐type electrode

The H_2_ concentration was monitored under anaerobic conditions using a Clark‐type Pt‐Ag/AgCl electrode chamber (DW1/AD; Hansatech, King's Lynn, UK) connected to a homemade polarographic box. Experimental cultures were harvested, resuspended in fresh Z8x to 3–4 μg ml^−1^ Chl*a*, and sparged with N_2_ or Ar for 30 min in the dark to achieve anaerobic conditions. Cultures were incubated under the corresponding atmosphere for another 2 h in the dark at 25°C. The H_2_ concentration was monitored for 6 min under actinic light (800 μmol photons m^−2^ s^−1^). The H_2_ production rates were calculated using linear regression.

### Nitrogenase activity assay

An acetylene reduction assay was used to determine nitrogenase activity as described previously (Leino *et al*., 2014). Filaments in sealed vials were flushed with Ar and supplemented with 10% acetylene in the headspace. Vials were kept for 20 h under 50 μmol photons m^−2^ s^−1^ at 30°C with agitation (120 rpm). The ethylene content in 20 μl of sample from the headspace was analysed using a gas chromatograph with a Carboxen^®^‐1010 PLOT Capillary Column and FID detector. Enzyme activity was calculated from the peak area and normalized to total protein content.

### Chl*a* and total sugar determination

Chl*a* concentration was determined in 90% methanol according to the method described by Meeks & Castenholz (1971). The total sugar content (simple sugars, oligosaccharides, and polysaccharides) was obtained using the colorimetric method as described by Dubois *et al*. (1956).

### Protein extraction and immunoblotting

Total protein was extracted as described previously (Zhang *et al*., 2009). Electrophoresis with loading based on equal protein content and immunoblotting were performed as described by Mustila *et al*. (2016). Specific antibodies raised against Flv3A (Agrisera, Vännäs, Sweden), NdhK (Agrisera), NifH (Agrisera), and HupL (provided by P. Tamagnini) were used.

### RNA isolation and reverse transcription quantitative polymerase chain reaction (RT‐qPCR) analysis

Isolation of total RNA, reverse transcription and qPCR analysis were performed as described previously (Ermakova *et al*., 2013). The *rnpB* gene was used as a reference for normalization. Primer pairs are listed in Table S1.

## Results

### Phenotypic characterization of *Anabaena* mutants deficient in Flv1A and Flv3A

**Fig. 1:**
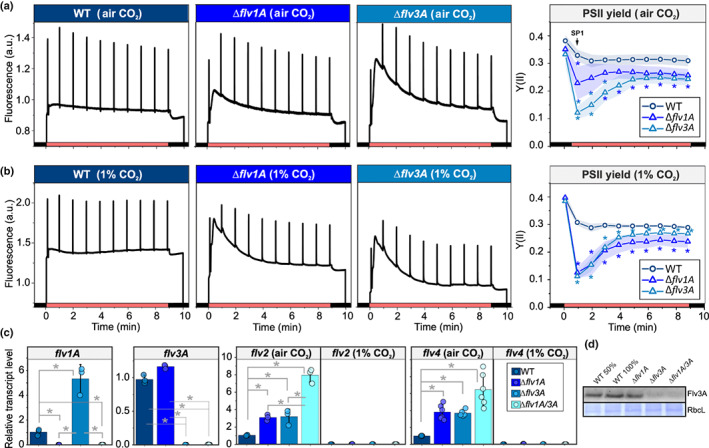
Fluorescence and transcript analysis of the diazotrophic *Anabaena* wild‐type (WT) and flavodiiron protein (FDP) mutant strains. (a) Representative Chl*a* fluorescence traces from filaments cultivated under air (0.04% CO_2_; LC) or (b) under 1% CO_2_ (HC) conditions. Filaments were dark acclimated for 10 min before illumination with 50 μmol photons m^−2^ s^−1^ of actinic light. The effective yield of PSII (Y(II)) was determined via the application of saturating pulses during induction curve measurements. Values are means ± SE; *n* = 3–4 biological replicates. (c) Analyses of FDP transcript levels as determined by reverse transcription quantitative polymerase chain reaction. Transcript abundances of *flv1A*, *flv3A*, *flv2* and *flv4* grown under LC and HC conditions are shown relative to the WT levels. The *flv2* and *flv4* levels under HC conditions are normalized to the respective WT levels under LC conditions to create a meaningful comparison. Values are means ± SD, from 3 or 6 (*flv4* LC) biological replicates, with individual replicate data shown as circles. Asterisks indicate statistically significant differences according to Student's *t*‐test (*, *P* < 0.05). (d) Immunodetection of Flv3A protein from diazotrophic filaments grown under LC conditions. A Coomassie blue‐stained band of the large subunit of Rubisco (RbcL) is shown as a loading control. A representative blot of three biological replicates is shown. See Fig. S7(d) for quantification. Asterisks indicate statistically significant differences according to Student's *t*‐test (*, *P* < 0.05). a.u., artificial units; SP, saturating pulse.

To investigate the function of the vegetative cell‐specific Flv1A and Flv3A proteins in diazotrophic *Anabaena* filaments, we used ∆*flv1A* and ∆*flv3A* deletion mutants (Fig. S1; Allahverdiyeva *et al*., 2013). The RT‐qPCR analysis confirmed the absence of *flv1A (all3891)* and *flv3A (all3895)* transcripts in ∆*flv1A* and ∆*flv3A*, respectively (Fig. 1b,c). An elevated *flv1A* transcript level was detected in ∆*flv3A*. Similar to SynFlv1 and SynFlv3, the AnaFlv1A and AnaFlv3A proteins are indispensable for diazotrophic and nondiazotrophic growth of *Anabaena* filaments under severe fluctuating light conditions in both LC and HC conditions (Fig. S4a; Allahverdiyeva *et al*., 2013).

Under constant light (50 μmol photons m^−2^ s^−1^), there were no significant differences in the growth of these mutants compared to WT, as measured by OD_750_ or Chl*a* concentration (Table 1; Fig. S4b). Total protein and sugar content of WT and ∆*flv1A* and ∆*flv3A* filaments were also similar (Table 1). Light micrographs of filaments indicated that all strains had a similar ratio of vegetative cells to heterocysts (Table 1), and no visible changes were observed in heterocyst morphology (Fig. S5).

**Table 1:** Growth characteristics and photosynthetic parameters of the *Anabaena* wild‐type (WT), ∆*flv1A*, and ∆*flv3A* filaments.

Parameters	Wild‐type	∆*flv1A*	∆*flv3A*
OD_750_	1.46 ± 0.18	1.45 ± 0.13	1.43 ± 0.18
Chl*a* (μg ml^−1^)	7.01 ± 0.80	6.66 ± 0.76	6.97 ± 0.96
Total protein (μg ml^−1^)	263.6 ± 31.7	265.3 ± 10.1	254 ± 14.1
Total sugars (μg ml^−1^)	53.8 ± 12.7	45.4 ± 4.9	43.9 ± 5.8
Heterocyst frequency (%)	12.7 ± 1.1	12.1 ± 0.9	10.7 ± 2.1
*F*_v_/*F*_m_ (with 10 μM DCMU)	0.45 ± 0.05	0.5 ± 0.01	0.49 ± 0.01
*F*_o_	0.91 ± 0.01	0.86 ± 0.00*	0.84 ± 0.00*
*F*_m_^D^	1.39 ± 0.02	1.25 ± 0.02*	1.26 ± 0.01*
*F*_m_′ (SP1)	1.46 ± 0.05	1.42 ± 0.04	1.49 ± 0.04
1‐qL (SP1)	0.20 ± 0.16	0.49 ± 0.34	0.62 ± 0.21*
qT	0.03 ± 0.01	0.13 ± 0.01*	0.21 ± 0.02*

Experimental cultures were grown under diazotrophic conditions in air (0.04% CO_2_; LC conditions) for 4 d. OD_750_, optical density at 750 nm; *F*_v_/*F*_m_, maximum quantum yield of PSII; *F*_o_, minimal level of fluorescence; *F*_m_^D^, maximal fluorescence in darkness; *F*_m_′, maximal fluorescence; 1‐qL, redox state of the plastoquinone pool (calculated as (*F*_m_′ − *F*_s_/*F*_m_′ − *F*_o_′) × *F*_o_′/*F*_s_ (Kramer *et al*., 2004)); qT, quenching due to state transition (calculated as (*F*_m_′ − *F*_m_^D^)/*F*_m_^D^ (Jensen *et al*., 2000)). Values are means ± SD; *n* = 3–5 biological replicates. Asterisks indicate statistically significant differences compared to the wild‐type, according to Student's *t*‐test (*, *P* < 0.05).

### Chl*a* fluorescence and P700 and ferredoxin redox changes reveal differential photosynthetic electron transport in ∆ *flv1A* and ∆ *flv3A*

Diazotrophic *Anabaena* WT, ∆*flv1A*, and ∆*flv3A* filaments were next subjected to analysis of Chl*a* fluorescence to determine the impact of Flv1A and Flv3A on photosynthetic electron transport in vegetative cells. Upon exposure to actinic light, maximal fluorescence (*F*_m_′) slightly increased from the dark‐adapted maximum (*F*_m_^D^), indicating a transition from state 2 to state 1 (Fig. 1a). The effective yield of PSII (Y(II)) remained stable during the illumination of WT filaments grown under LC (Fig. 1a) and HC (Fig. 1b) conditions.

Both ∆*flv1A* and ∆*flv3A* mutants showed significantly lower *F*_m_^D^ than the WT in LC (Fig. 1a; Table 1), as well as lower PSII peaks in 77 K fluorescence emission spectra after dark‐adaption (Fig. S6d; Methods S2), implying a more pronounced state 2 in the dark. Accordingly, a stronger state‐2‐to‐state‐1 transition (qT, Table 1) was observed during illumination in comparison to the WT, similar to the phenotype described previously in the *Synechocystis ∆flv3* mutant (Elanskaya *et al*., 2021). Notably, fluorescence kinetics during dark‐to‐light transitions were affected differently in the two mutants grown under LC conditions. Illumination of ∆*flv3A* filaments resulted in a rapid increase in fluorescence, which was gradually quenched but remained at a higher steady‐state level (*F*_s_) than in the WT or ∆*flv1A*. The ∆*flv1A* mutant showed only a moderate increase and then a gradual decay of fluorescence, reaching the WT *F*_s_ level after 4 min of illumination. Unlike LC‐grown filaments, ∆*flv1A* and ∆*flv3A* grown under HC conditions revealed a similar fluorescence increase during dark‐to‐light transitions, which gradually decayed and reached WT levels by the end of the period of illumination (Fig. 1b).

The effective yield of PSII decreased both in LC‐ (to 70% and 37% of WT level in ∆*flv1A* and ∆*flv3A* mutants, respectively) and HC‐grown filaments (to 42% and 37% of the WT level in Δ*flv1A* and Δ*flv3A* mutants, respectively) (Fig. 1a,b). After that, Y(II) gradually recovered over the course of the period of illumination, although the mutants did not reach WT levels (Fig. 1a,b). These Y(II) kinetics resemble those measured from FDP knockout mutants in *Synechocystis* (Helman *et al*., 2003; Nikkanen *et al*., 2020), *Chlamydomonas reinhardtii* (Chaux *et al*., 2017), the moss *Physcomitrium patens* (Gerotto *et al*., 2016), and in the liverwort *Marchantia polymorpha* (Shimakawa *et al*., 2017). Notably, the maximum quantum yield of PSII, *F*_v_/*F*_m_, did not differ significantly between the mutants and WT (Table 1).

The transient post‐illumination increase in fluorescence (F_0_ rise, Methods S2), which reflects NDH‐1 mediated reduction of the plastoquinone (PQ) pool in darkness (Mi *et al*., 1995), was higher in both ∆*flv1A* and ∆*flv3A* grown under LC and HC conditions (Fig. S6a,b). In line with the lower *F*_m_^D^ (Table 1), this suggests elevated electron flux into the PQ pool in the dark in ∆*flv1A* and ∆*flv3A* by NDH‐1. Considering that the abundance of NdhK, a core subunit of NDH‐1, was similar between all genotypes (Fig. S6c), the difference in F_0_ rise may be caused by an increase in the availability of reduced Fd, the electron donor to both FDPs and NDH‐1 (Nikkanen *et al*., 2021), or by post‐translational regulatory factors.

**Fig. 2:**
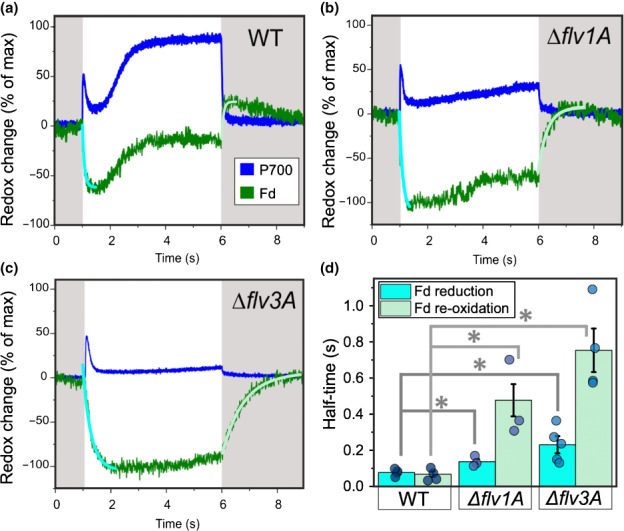
Redox changes of P700 and Fd upon dark–light–dark transitions in the diazotrophic *Anabaena* wild‐type (WT), Δ*flv1A*, and Δ*flv3A* filaments. Filaments were grown under an irradiance of 50 μmol photons m^−2^ s^−1^ and in air (0.04% CO_2_; LC conditions) for 4 d, harvested and adjusted to a Chl*a* concentration of 20 μg ml^−1^. P700 and Fd redox changes were then deconvoluted from the absorbance differences using specifically determined differential model plots (model spectra) for *Anabaena* (see the Materials and Methods section; Fig. S9a,b). Positive and negative changes indicate oxidation and reduction, respectively. Maximal levels of Fd reduction and P700 oxidation in each sample were used to normalize the traces. (a–c) Representative traces of four biological replicates are shown. (d) Half‐times of initial reduction and post‐illumination re‐oxidation of Fd as determined from first‐exponential fitting to the kinetics of the deconvoluted Fd signal (blue and light green curves in panels a–c). Values shown are means ± SE from 3 to 4 biological replicates, with individual data points shown as circles. Asterisks indicate statistically significant differences according to Student's *t*‐test (*, *P* < 0.05).

At the onset of high irradiance, both Δ*flv1* (Fig. S8) and Δ*flv3* mutants of *Synechocystis* are unable to rapidly re‐oxidize Fd, causing an accumulation of electrons at P700 (Nikkanen *et al*., 2020; Theune *et al*., 2021). To examine whether this occurs in *Anabaena* Δ*flv1A* and Δ*flv3A* mutants, we determined the high light‐induced redox changes in Fd and P700 from near‐infrared absorbance differences using the Dual KLAS/NIR spectrophotometer. The results indicated that, similar to *Synechocystis* Δ*flv3* (Nikkanen *et al*., 2020) and Δ*flv1* mutants (Fig. S8), both *Anabaena* mutants suffered from delayed re‐oxidation of Fd and P700 upon illumination (Fig. 2a–c), having significantly lowered post‐illumination re‐oxidation rates of Fd (Fig. 2d). Furthermore, significantly lower rates of initial Fd reduction upon illumination were measured in both mutants (Fig. 2d), likely due to decreases in initial electron transport from PSII (Fig. 1a). Unlike in *Synechocystis*, there was a clear difference between the two *Anabaena* mutants, with Δ*flv3A* exhibiting a more pronounced delay in the re‐oxidation of Fd than Δ*flv1A*. The differences in electron transport to and from PSI were not caused by differences in the ratio between the size of the reducible Fd pool and the size of the oxidisable P700 pool, as determined from maximal NIR absorbance changes attributable to Fd reduction and P700 oxidation (Fig. S9c).

### O_2_ and CO_2_ fluxes, and generation of proton motive force are disturbed in *Anabaena* FDP mutants

To clarify the specific impacts of *flv1A* and *flv3A* deletions on real‐time gas fluxes in diazotrophic filaments of *Anabaena*, we used MIMS. The MIMS technique combined with the use of the ^18^O_2_ isotopologue enabled us to distinguish between O_2_ uptake and photosynthetic O_2_ production.

**Fig. 3:**
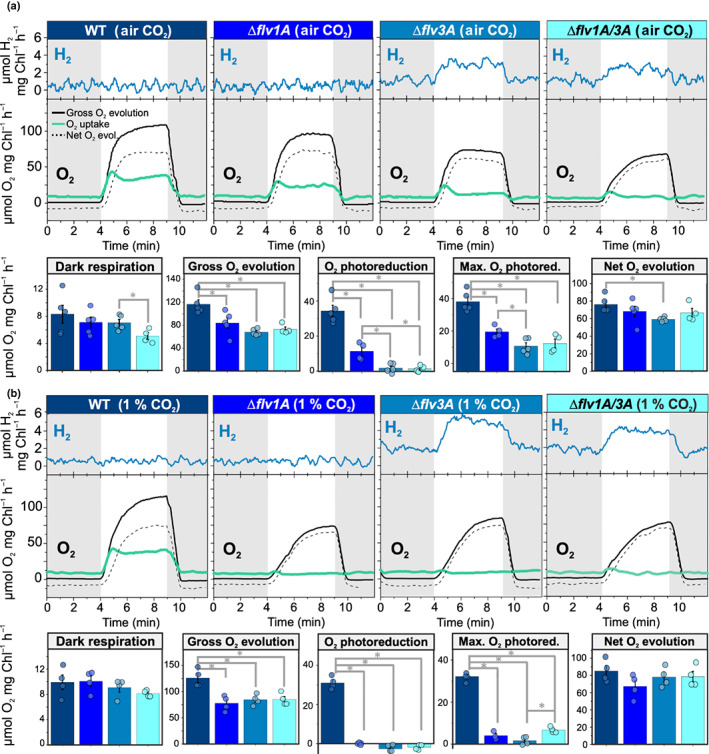
Oxygen (O_2_) and hydrogen (H_2_) exchange rates of diazotrophic *Anabaena* wild‐type (WT), ∆*flv1A*, ∆*flv3A*, and ∆*flv1A/3A* filaments, measured by membrane inlet mass spectrometry. The filaments were cultivated for 4 d in (a) air (0.04% CO_2_; LC) and (b) 1% CO_2_ (HC), after which the filaments were harvested and Chl*a* concentration was adjusted to 10 μg ml^−1^. Gas exchange was monitored for 4 min in darkness (grey areas of the graphs) followed by 5 min of high irradiance (white light at 500 μmol photons m^−2^ s^−1^) and for an additional 3 min in the dark. For LC measurements, samples were supplemented with 1.5 mM sodium bicarbonate (NaHCO_3_). The traces are representative of 4–5 independent biological replicates. The column charts in (a) and (b) show the rates for pre‐illumination dark respiration (determined from the average O_2_ uptake rate between 1 and 4 min into the experiment), gross O_2_ evolution (determined from the average steady‐state rate between 8 and 10 min), O_2_ photoreduction (determined from average steady‐state O_2_ uptake rate between 8 and 10 min – dark respiration rate), maximal O_2_ photoreduction (determined from the magnitude of the O_2_ uptake peak during the first minute of illumination – dark respiration rate), and net O_2_ evolution rate (calculated as the difference between the rates of gross O_2_ evolution and O_2_ uptake). Values for the columns are averages of 4–5 biological replicates ± SE, with individual data points shown as circles. Asterisks indicate statistically significant differences according to Student's *t*‐test (*, *P* < 0.05).

Illumination of WT filaments grown under LC demonstrated a rapid increase in O_2_ uptake rate. This fast induction phase was followed by a decay that stabilized after 3 min (Fig. 3a). This pattern resembles previously described triphasic kinetics of O_2_ photoreduction in *Synechocystis* grown under LC conditions (Santana‐Sánchez *et al*., 2019), and in HC‐grown *C. reinhardtii* cells illuminated with high light intensity (Saroussi *et al*., 2019; Burlacot *et al*., 2020a). While all mutants showed slight but statistically nonsignificant decreases in O_2_ uptake rates in darkness (Fig. 3a), the rate of O_2_ consumption under illumination was affected to different extents in ∆*flv1A* and ∆*flv3A* filaments. The ∆*flv3A* mutant exhibited strong impairment of light‐induced O_2_ uptake, showing a maximal rate that was 28% of the WT level at the onset of light, declining to a residual rate of 5% of the WT rate by the end of the period of illumination. In contrast to the *Synechocystis* Δ*flv1* mutant, in which O_2_ photoreduction is almost fully eliminated (Fig. S10), the *Anabaena* ∆*flv1A* filaments showed an intermediate phenotype with a maximum light‐induced O_2_ reduction rate of 52% of the WT level, which declined to 33% of the WT level (Fig. 3a). These results suggested that both AnaFlv1A and AnaFlv3A contribute to the Mehler‐like reaction, but to differing extents and presumably in different homo‐/hetero‐oligomeric arrangements.

To clarify whether the homo‐oligomers of AnaFlv1A in ∆*flv3A* and, conversely, homo‐oligomers of AnaFlv3A in ∆*flv1A* contribute to the observed O_2_ photoreduction rates (Fig. 3a), we constructed a double mutant ∆*flv1A*/*3A* (Figs S1d,e, S11). Concomitant inactivation of both *flv1A* and *flv3A* strongly inhibited O_2_ photoreduction under LC (Fig. 3a). Previous studies with *Synechocystis* cells (Zhang *et al*., 2009; Eisenhut *et al*., 2012; Santana‐Sánchez *et al*., 2019) and nondiazotrophic *Anabaena* WT filaments (Ermakova *et al*., 2013) showed high transcript abundances of *flv2* and *flv4* under LC conditions. Therefore, we next investigated the abundance of *flv2* and *flv4* transcripts in diazotrophic *Anabaena* filaments grown under LC and HC conditions using RT‐qPCR. The ∆*flv1A* and ∆*flv3A* mutants grown under LC conditions demonstrated significantly higher *flv2* and *flv4* transcript levels compared to the WT (Fig. 1c). Under HC conditions, transcript abundances of *flv2* and *flv4* were drastically lower in all genotypes compared to LC conditions (Fig. 1c). This prompted us to examine the possible contributions of AnaFlv2 and AnaFlv4 proteins to the Mehler‐like reaction by comparing the O_2_ photoreduction rates in ∆*flv1A* and ∆*flv3A* mutants grown under LC (Fig. 3a) vs HC conditions (Fig. 3b), where the expression of *flv2* was induced and the expression of *flv4* was repressed.

While the O_2_ photoreduction observed in WT filaments grown under HC conditions was comparable to that observed in WT filaments grown under LC conditions (Fig. 3a), the inactivation of *flv1A* and/or *flv3A* fully eliminated light‐induced O_2_ reduction in filaments grown under HC conditions (Fig. 3b). This suggests that the highly expressed *flv2* and *flv4* contribute to O_2_ photoreduction in diazotrophic ∆*flv1A* and ∆*flv3A* filaments grown under LC conditions. In order to investigate the potential involvement of the *Anabaena* orthologue of Plastid terminal oxidase (PTOX) (McDonald *et al*., 2003) in catalysing the residual O_2_ photoreduction in ∆*flv1A*, we measured the O_2_ exchange in the mutant in the presence of *n*‐propyl gallate (nPG), an inhibitor of PTOX. While some inhibition of total O_2_ flux was detected, O_2_ photoreduction was largely unaffected by nPG (Fig. S12a–c), suggesting it to be independent of PTOX. A significantly elevated transcript level of *ptox* was detected in ∆*flv3A*, while ∆*flv1A* showed no difference from the WT (Fig. S12d).

**Fig. 4:**
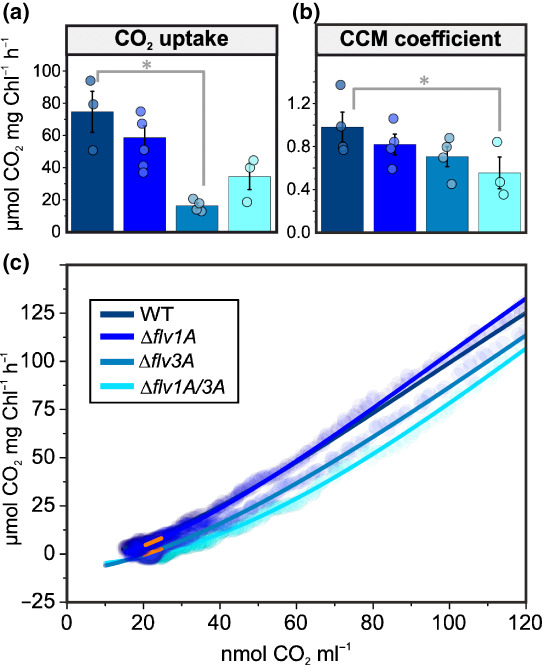
Carbon dioxide exchange parameters in diazotrophic *Anabaena* wild‐type (WT), ∆*flv1A*, ∆*flv3A*, and ∆*flv1A/3A* filaments. (a) Steady‐state CO_2_ uptake rates in *Anabaena* filaments grown in air (0.04% CO_2_; LC conditions). Measurements were performed by membrane inlet mass spectrometry simultaneously with the O_2_ measurements in Fig. 3(a) and the experimental setup was as described in the legend for Fig. 3. The CO_2_ uptake rate was calculated as the average rate between 4 and 5 min after the onset of illumination. (b, c) The carbon‐concentrating mechanism (CCM) coefficient in diazotrophic *Anabaena* WT and mutant filaments cultivated for 4 d under LC conditions, after which the filaments were harvested and the Chl*a* concentration was adjusted to 10 μg ml^−1^. Cells were illuminated with 500 μmol photons m^−2^ s^−1^ for *c*. 30–45 min or until the CO_2_ consumption rate was close to zero due to C_i_ depletion in the sample. The CO_2_ fixation rate was then plotted as a function of CO_2_ concentration (c) and fitted to a modified Hill equation. Finally, the slope of a linear fit between 20 and 25 nmol CO_2_ ml^−1^ (orange line) was used to determine the CCM coefficient (Douchi *et al*., 2019). The values in (a, b) are averages from 3 to 5 biological replicates ± SE, with the individual data points shown as circles. Asterisks indicate statistically significant differences according to Student's *t*‐test (*, *P* < 0.05).

The net photosynthetic O_2_ production rate under LC conditions in ∆*flv1A* was comparable to that observed in the WT, but in ∆*flv3A* it was lower (Fig. 3a). Moreover, while the steady‐state CO_2_ fixation rates of all deletion mutants were diminished compared to the WT, ∆*flv3A* showed significant impairment in comparison to ∆*flv1A* (Fig. 4a). The initial peak in CO_2_ uptake rate, which has been suggested to derive from CCM activation in *Synechocystis* (Liran *et al*., 2018), was also lower in both mutant strains than in the WT (Fig. S13b). It was recently reported that the FDPs are required for the activation of CCM in *C. reinhardtii* (Burlacot *et al*., 2022). In order to ascertain whether the same is true in *Anabaena*, we determined the CCM coefficient (Douchi *et al*., 2019) by measuring CO_2_ fixation rates in CO_2_‐limiting conditions, where CCM activity becomes the limiting factor for carbon fixation (Fig. 4b,c). Indeed, CCM activity was decreased in all mutant strains, and significantly in ∆*flv1A/3A* (Fig. 4b).

**Fig. 5:**
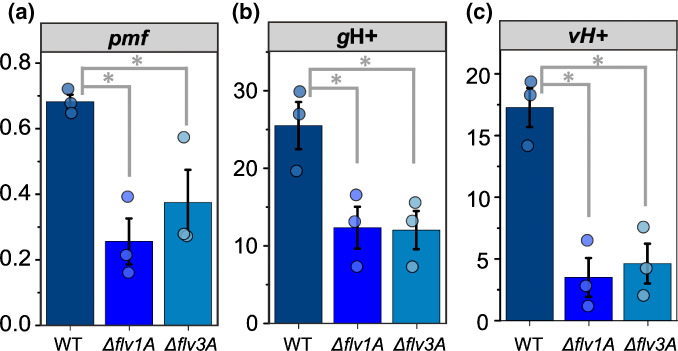
Analysis of the proton motive force (pmf) during the onset of high irradiance in diazotrophic *Anabaena* wild‐type (WT), ∆*flv1A*, and ∆*flv3A* filaments. The filaments were cultivated for 4 d in air (0.04% CO_2_; LC conditions), after which they were harvested and the Chl*a* concentration was adjusted to 7.5 μg ml^−1^. Samples were illuminated with 500 μmol photons m^−2^ s^−1^ of green light for 2 min, during which the post‐illumination relaxation kinetics of the electrochromic shift (ECS) signal (absorbance difference between 500 and 480 nm) were monitored at specific time points using a JTS‐10 spectrophotometer. Dark interval relaxation kinetic data after 27 s of illumination are shown here; see Fig. S3 for the full results of the 2 min experiment. (a) The magnitude of the pmf after 27 s of illumination was determined as the light‐induced change in the ECS signal. (b) The conductivity of the thylakoid membrane (the *g*H+ parameter, which mostly depends on the activity of ATP synthase) was determined as the inverse of the time constant of a first‐order fit to the relaxation kinetics of the ECS signal. (c) Proton flux (*v*H+) was calculated as pmf × *g*H+. The values in panels (a–c) are means from three independent biological replicates ± SE, with individual data points shown as circles. Asterisks indicate statistically significant differences according to Student's *t*‐test (*P* < 0.05).

In *C. reinhardtii*, the dependence of CCM activation on FDPs is a result of the contribution of FDPs to the generation of pmf over the thylakoid membrane (Burlacot *et al*., 2022). As SynFlv1 and SynFlv3 have been shown to have a crucial role in the generation of pmf during dark‐to‐light transitions (Nikkanen *et al*., 2020), comparable to that of FLVA/B in *P. patens* (Gerotto *et al*., 2016) and *C. reinhardtii* (Chaux *et al*., 2017), we proceeded to investigate how the generation of pmf is affected in *Anabaena* ∆*flv1A* and ∆*flv3A* mutants by measuring the DIRK of the ECS signal during dark‐to‐high‐irradiance transitions (Figs 5, S3). Similar to the aforementioned organisms, both ∆*flv1A* and ∆*flv3A* had a significantly impaired ability to generate pmf upon transitions to high irradiance (Fig. 5a). This was due to a diminished proton flux to the lumen (*v*H+) (Fig. 5c), which was partially compensated for by a lowered conductivity of the thylakoid membrane (*g*H+) (Fig. 5b).

Under HC conditions, both ∆*flv1A* and ∆*flv3A* mutants had lower gross O_2_ evolution relative to WT (Fig. 3b), and a delay in the induction of O_2_ evolution upon illumination (Fig. S13e). While under LC ∆*flv1A* and ∆*flv1A*/*3A* showed significantly longer gross O_2_ evolution induction half‐times than ∆*flv3A* (Figs 3a, S13d), under HC all three mutant strains were similarly impaired (Figs 3b, S13e), suggesting that AnaFlv1A also possesses an AnaFlv3A‐independent, LC‐specific function.

### Consequences of *flv1A* or *flv3A* deletion on diazotrophic metabolism

The results above indicate that AnaFlv1A and AnaFlv3A impact bioenergetics in vegetative cells to different extents in LC‐grown diazotrophic *Anabaena*. Previous studies with different diazotrophic *Anabaena* species have demonstrated that disruption of PSII activity in vegetative cells has implications for N_2_ and H_2_ metabolism inside heterocysts (Khetkorn *et al*., 2012; Chen *et al*., 2014). We therefore examined whether the absence of AnaFlv1A or AnaFlv3A from vegetative cells impacts heterocyst metabolism. To this end, we analysed the nitrogenase activity and H_2_ fluxes of diazotrophic *Anabaena* WT, ∆*flv1A* and ∆*flv3A* filaments.

**Fig. 6:**
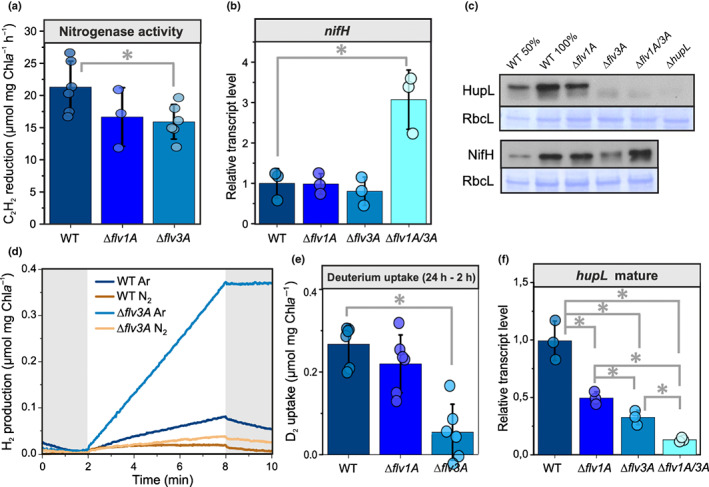
Hydrogen (H_2_) metabolism of diazotrophic filaments of *Anabaena* wild‐type (WT) and flavodiiron protein (FDP) mutants. (a) Nitrogenase activity was measured using the acetylene reduction assay. (b) Relative transcript levels of mature *nifH*. (c) Immunodetection of the HupL and NifH proteins with specific antibodies from cells grown in air (0.04% CO_2_; LC conditions). Coomassie blue‐stained bands of the large Rubisco subunit are shown as loading controls. (d) Net H_2_ yield was monitored using a H_2_ electrode under an argon (Ar) or nitrogen (N_2_) atmosphere in darkness (grey areas) and under an irradiance of 800 μmol photons m^−2^ s^−1^. (e) Deuterium (D_2_) uptake by the filaments was calculated from the difference in D_2_ concentration between 2h and 24 h after the injection in the vials initially flushed with Ar. (f) Relative transcript levels of the mature *hupL*. Values in panels (a), (b), (e) and (f) are means ± SD from three biological replicates, with individual data points shown as circles. Asterisks indicate statistically significant differences according to Student's *t*‐test (*, *P* < 0.05).

Both ∆*flv1A* and ∆*flv3A* showed somewhat lower nitrogenase activity in comparison to WT, yet this decrease was significant in ∆*flv3A* only (Fig. 6a). This finding in ∆*flv3A* was likely due to a lowered nitrogenase content, as indicated by the reduced amount of the NifH subunit at the protein (but not transcript) level (Figs 6b,c, S7d). By contrast, significantly elevated NifH transcript levels and slightly elevated NifH protein levels were detected in ∆*flv1A/3A* filaments (Figs 6b,c, S7d). Real‐time gas exchange monitored by MIMS revealed no changes in the H_2_ concentration in the WT and ∆*flv1A* during dark–light transitions. By contrast, the ∆*flv3A* mutant, as well as the ∆*flv1A/3A* double mutant, showed increased H_2_ levels in the dark and clear light‐induced H_2_ production in heterocysts (Fig. 3a). This result was confirmed by a second independent ∆*flv3A* mutant strain showing similar light‐induced H_2_ production (Fig. S13a). Interestingly, the ∆*flv3A* and ∆*flv1A/3A* mutants cultivated under HC conditions demonstrated even higher H_2_ photoproduction rates (Fig. 3b). Although ∆*flv3A* and ∆*flv1A/3A* filaments showed real‐time H_2_ production under oxic conditions, the rate of H_2_ production remained low.

Next, we monitored H_2_ in anoxic cultures using a Clark‐type electrode. Under an N_2_ atmosphere, ∆*flv3A* exhibited a significantly higher light‐dependent net H_2_ yield (Fig. 6d) and a three‐fold higher H_2_ production rate compared to the WT (Fig. S7a). To confirm that the H_2_ production is nitrogenase‐mediated, we monitored the reaction under an Ar atmosphere, as in the absence of N_2_ substrate, nitrogenase reduces protons to H_2_. Indeed, the specific H_2_ photoproduction rate of WT filaments under Ar was *c*. 7‐fold higher compared to the N_2_ atmosphere (Fig. S7a). In ∆*flv3A*, net H_2_ yield was strongly enhanced under Ar, and the production rate increased *c*. 10‐fold compared to N_2_ (Fig. S7a). A drastically decreased transcript abundance of *hoxH* in both ∆*flv1A* and ∆*flv3A* mutants compared to WT (Fig. S7b) implied a negligible contribution of Hox to H_2_ production in ∆*flv3A*. These results provide evidence that the enhanced H_2_ production in the ∆*flv3A* mutant is mediated by nitrogenase.

However, the increase in net H_2_ photoproduction in ∆*flv3A* (Fig. 3a) did not correlate with the decrease in nitrogenase activity (Fig. 6a) or the abundance of NifH (Fig. 6c). This is in line with reports of low nitrogenase activity in ∆*hupL* (Happe *et al*., 2000; Leino *et al*., 2014). The net nitrogenase‐mediated production of H_2_ in heterocysts is strongly affected by the activity of uptake hydrogenase (Hup), which oxidizes H_2_ (Tamagnini *et al*., 2007). It is therefore conceivable that impairment of Hup function could account for the increased production of H_2_ in ∆*flv3A*, comparable to ∆*hupL* (Fig. S14). To examine the H_2_ fluxes, we traced the uptake of deuterium (^2^H_2_, D_2_) by WT, ∆*flv1A*, and ∆*flv3A* strains. Whilst WT and ∆*flv1A* filaments efficiently consumed D_2_, ∆*flv3A* showed a significantly lower capacity for D_2_ uptake (Fig. 6e). These observations confirmed that the impaired capacity of ∆*flv3A* to recycle H_2_ likely explains the increased net H_2_ yield in the mutant. Indeed, we detected a significant downregulation of the mature form of the *hupL* transcript in ∆*flv1A*, and an even more pronounced downregulation was observed in ∆*flv3A* (Fig. 6f). Moreover, immunoblotting revealed a lack of detectable HupL protein in ∆*flv3A* and ∆*flv1A/3A*, comparable to findings for the ∆*hupL* mutant (Figs 6c, S7d), which, similar to ∆*flv3A*, showed enhanced net H_2_ yield (Fig. S7). Interestingly, an increased transcript level of the heterocyst‐specific ferredoxin (*fdxH*), which functions as electron donor to nitrogenase (Magnuson, 2019), was detected in ∆*flv3A* and ∆*flv1A/3A* (Fig. S7c).

## Discussion

Heterocyst‐forming cyanobacteria are considered one of the earliest forms of multicellular filaments. Despite extensive characterization of heterocyst differentiation, little is known about the co‐regulation and interdependence of N_2_ fixation in heterocysts and oxygenic photosynthesis in vegetative cells. Under challenging environmental conditions, diazotrophic cyanobacteria must find a balance between photochemical reactions and downstream processes that consume electrons in both cell types. In this study, we used ∆*flv1A* and ∆*flv3A* mutants of *Anabaena* to examine the physiological significance of the vegetative cell‐specific AnaFlv1A and AnaFlv3A proteins in the bioenergetic processes of diazotrophic cyanobacteria. We have demonstrated that both AnaFlv1A and AnaFlv3A proteins, presumably as hetero‐oligomers, are required for efficient induction of the Mehler‐like reaction and, consequently, for efficient generation of pmf, and likely for activation of CCM during dark‐to‐light transitions, making FDPs crucial for growth when light intensity rapidly fluctuates. Moreover, AnaFlv3A exhibits an important link to H_2_ metabolism inside the heterocyst, as inactivation of this protein results in high H_2_ photoproduction even under ambient air.

### In the absence of AnaFlv1A, AnaFlv3A cooperates with AnaFlv2 and/or AnaFlv4 to mediate O_2_ photoreduction under LC conditions

In line with previous results showing a decrease in the expression of both *flv1A* and *flv3A* in *Anabaena* WT upon shifts to diazotrophic conditions (Ermakova *et al*., 2013), single deletions of AnaFlv1A or AnaFlv3A did not affect the diazotrophic growth of mutants under continuous illumination (Table 1). However, both the AnaFlv1A and AnaFlv3A proteins are indispensable during sudden changes in light intensity, similar to their homologous proteins in other species (Fig. S4a; Allahverdiyeva *et al*., 2013; Gerotto *et al*., 2016; Jokel *et al*., 2018). Here, we have demonstrated that when both AnaFlv1A and AnaFlv3A proteins are expressed in WT filaments, the rate of the Mehler‐like reaction is rapidly increased during dark‐to‐light transitions, likely due to the activity of AnaFlv1A/Flv3A hetero‐oligomers (Fig. 3). Accordingly, the absence of either AnaFlv1A or AnaFlv3A impairs O_2_ photoreduction (Fig. 3a), resulting in a strong reduction of the PQ pool upon illumination (Fig. 1a, 1‐qL parameter in Table 1), a decrease in PSII yield (Fig. 1a), and impairment of PSI and Fd oxidation (Fig. 2). This phenotype is exaggerated in the mutant lacking AnaFlv3A, which showed a stronger state‐2‐to‐state‐1 transition and a more severely limited ability to oxidize PSI than the mutant lacking AnaFlv1A (Figs 1, 2; Table 1).

In contrast to the *Synechocystis* Δ*flv1* mutant (Fig. S10), AnaFlv3A can promote O_2_ photoreduction in *Anabaena* Δ*flv1A* (Fig. 3a), resulting in only a 67% inhibition of steady‐state O_2_ photoreduction under LC growth conditions (Fig. 3a). The near elimination of steady‐state O_2_ photoreduction in the Δ*flv1A/flv3A* double mutant under LC conditions (Fig. 3a) and in the single mutants under HC conditions (Fig. 3b) (where AnaFlv2 and AnaFlv4 are strongly downregulated) prompts us to propose functional AnaFlv3A/Flv2‐4 oligomerization, and/or cooperation between a AnaFlv3A/Flv3A homo‐oligomer and AnaFlv2/Flv4 (homo)hetero‐oligomers. Accordingly, the strong impairment of O_2_ photoreduction in Δ*flv3A* might be due to the inability of AnaFlv1A to function as a homo‐oligomer and/or cooperate with AnaFlv2/Flv4. It is worth emphasising that both ∆*flv1A* and ∆*flv3A* mutants showed similarly enhanced accumulation of *flv2* and *flv4* transcripts (Fig. 1c). As the ∆*flv3A* mutant showed an elevated *flv1A* transcript level (Fig. 1c), the inhibition of O_2_ photoreduction in ∆*flv3A* cannot be due to downregulation of other FDPs. No contribution of SynFlv3/Flv3 homo‐oligomers in the Mehler‐like reaction was observed *in vivo* (Mustila *et al*., 2016), contrary to findings from *in vitro* studies which suggested that SynFlv3/Flv3 homo‐oligomers function in NAD(P)H‐dependent O_2_ reduction (Vicente *et al*., 2002; Brown *et al*., 2019). Instead, a possible photoprotective function of SynFlv3/Flv3 homo‐oligomers via an unknown electron transport network has been proposed (Mustila *et al*., 2016). In *Anabaena* ∆*flv1A*, AnaFlv3A/Flv3A homo‐oligomers may be involved in controlling cation homeostasis, which in turn may affect the reversible association of AnaFlv2/Flv4 hetero‐oligomers with the thylakoid membrane (Zhang *et al*., 2012) and, consequently, their involvement in O_2_ photoreduction. Another possibility is the involvement of AnaFlv3A/Flv3A homo‐oligomers in the reduction of nitric oxide (NO), similar to the FDP‐dependent photoreduction of NO in *C. reinhardtii* (Burlacot *et al*., 2020b). As NO has a strong inhibitory effect on PSII activity (Solymosi *et al*., 2022), its efficient reduction by FDPs could serve a photoprotective or regulatory role. Overall, our results suggest a role for AnaFlv3A in promoting steady‐state O_2_ photoreduction under diazotrophic LC conditions in an AnaFlv2/Flv4‐dependent manner. Moreover, under LC but not HC conditions, the lack of both AnaFlv1A and AnaFlv3A resulted in a more severe delay in the induction of O_2_ evolution during dark‐to‐light transition in comparison to the lack of AnaFlv3A only (Fig. S13d,e). This suggests that AnaFlv1A may also function independently of AnaFlv3A in an unknown role that facilitates photosynthetic electron transport under LC. Understanding the potential functions of AnaFlv2 and/or AnaFlv4 in these processes and their interactions with AnaFlv1A and AnaFlv3A requires further investigation.

All *Anabaena* FDP mutants studied here exhibited reduced CCM activity, as deduced from slightly lowered CCM coefficients (albeit significantly in the double mutant only) (Fig. 4b,c), as well as lowered initial peaks in CO_2_ uptake rate during dark‐to‐light transitions (Fig. S13b,c). Moreover, steady‐state CO_2_ uptake was severely diminished in ∆*flv3A* in comparison to the WT (Fig. 4a). This may result from impaired energization of CCM in the absence of AnaFlv3A. The pmf generated by FDPs and cyclic electron transport (CET) has been recently shown to be important for inducing and maintaining CCM activity in *C. reinhardtii* (Burlacot *et al*., 2022). As both ∆*flv1A* and ∆*flv3A* exhibited significantly impaired pmf generation upon dark‐to‐light transitions (Fig. 5a), we hypothesize that the AnaFlv1A/Flv3A hetero‐oligomer is required to rapidly induce the Mehler‐like reaction, which is important for the generation of pmf and induction of CCM activity during dark‐to‐light transitions. The molecular mechanism of the FDP‐dependency of the CCM requires further investigation, however, as the mechanisms of CCM differ between algae and cyanobacteria (Long *et al*., 2016). Most importantly, cyanobacteria lack the bestrophin‐like HCO_3_^−^ transporters whose energization was recently shown to depend on FDPs and CET in *C. reinhardtii* (Mukherjee *et al*., 2019; Burlacot *et al*., 2022). However, operation of the plasma membrane Na^+^/HCO_3_^−^ antiporters SbtA and BicA in cyanobacterial CCMs depend on the export of Na^+^ from the cytosol by the NhaS3 Na^+^/H^+^ antiporter (Long *et al*., 2016). Along with the light‐dependent plasma membrane‐localized H^+^ exchanger PxcA (Sonoda *et al*., 1998), FDPs may be necessary to energize NhaS3‐dependent Na^+^ import by consuming H^+^ in the cytosol (thus increasing pmf). Moreover, the HCO_3_^−^ transporter BCT1 consumes ATP to import HCO_3_^−^ (Long *et al*., 2016), suggesting that efficient activation of the ATP synthase by pmf generation is required for CCM induction in cyanobacteria. In both *Anabaena* ∆*flv1A* and ∆*flv3A* (Figs 5b, S3b), as well as in *Synechocystis* ∆*flv3* (Nikkanen *et al*., 2020), the activity of the ATP synthase was diminished during dark‐to‐light transitions, which may impair CCM induction.

Compelling evidence was recently provided for coordination and functional redundancy between NDH‐1 and Flv1/Flv3, jointly contributing to efficient oxidation of PSI in *Synechocystis* (Nikkanen *et al*., 2020) and in *P. patens* (Storti *et al*., 2020a,b). NDH‐1‐mediated CET in *Anabaena* could also partially compensate for a lack of AnaFlv1A and AnaFlv3A, as evidenced by observations of a stronger F_0_ rise in both mutants (Fig. S6a). Unlike *Synechocystis* cells, *Anabaena* filaments also express orthologs of PTOX (McDonald *et al*., 2003), which in *C. reinhardtii* and vascular plants functions as an electron valve from plastoquinol to O_2_, thereby controlling the redox state of the PQ pool (Houille‐Verges *et al*., 2011; Saroussi *et al*., 2019). However, as the addition of the PTOX inhibitor nPG did not eliminate the residual O_2_ photoreduction in Δ*flv1A* (Fig. S12a–c), it is unlikely that AnaPtox is a major contributor to it.

### Inactivation of AnaFlv3A leads to enhanced H_2_ yield in heterocysts even under oxic conditions

Elevated photoproduction of H_2_ in diazotrophic filaments lacking vegetative cell‐specific AnaFlv3A under oxic (Fig. 3) and microoxic conditions (Fig. 6) demonstrated bioenergetic interdependence between vegetative cells and heterocysts. The heterocyst‐originated production of H_2_ in ∆*flv3A* was rapidly induced upon exposure to light and occurred concomitantly with O_2_ evolution in vegetative cells (Fig. 3). Moreover, the rate of H_2_ photoproduction in ∆*flv3A* responded positively to an increase in CO_2_ availability (Fig. 3b).

In the absence of N_2_, the main substrate for nitrogenase, all electrons can be directed to H_2_ production (Hoffman *et al*., 2014; Wilson *et al*., 2021) allowing a less costly reaction, whereby only 4 mol of ATP are required to produce 1 mol of H_2_. Removal of the N_2_ substrate (by replacement with Ar) led to a 10‐fold increase in the H_2_ photoproduction rate in ∆*flv3A*, demonstrating nitrogenase‐dependent H_2_ photoproduction (Fig. 6d). A recent report suggested that overexpressing heterocyst‐specific Flv3B leads to more stable microoxic conditions inside the heterocysts, notably increasing the H_2_ production yield, presumably *via* the bidirectional hydrogenase Hox (Roumezi *et al*., 2020). In contrast to the unidirectional production of H_2_ by nitrogenase, Hox catalyses the reversible reduction of protons to H_2_ (Bothe *et al*., 2010). We do not consider the contribution of Hox to the photoproduction of H_2_ by the ∆*flv3A* mutant, as the net production does not fit the bidirectional nature of the enzyme. Observations of the significant downregulation of *hoxH* transcripts in ∆*flv3A* (Fig. S7b) further support this assumption. Altogether, these results indicate that the increased light‐induced H_2_ photoreduction in ∆*flv3A* is mediated by nitrogenase activity.

Strikingly, the increase in H_2_ photoproduction yield in ∆*flv3A* was due to significant downregulation of HupL, the large subunit of the uptake hydrogenase (Fig. 6c,f). The absence of functional Hup suppressed the H_2_ recycling pathway (Fig. 6e) and caused a release of H_2_ from heterocysts of ∆*flv3A* filaments (Figs 3, 6). Although the Hox hydrogenase can also potentially contribute to H_2_ recycling, its input to the process is unclear. Despite *hoxH* transcripts in all mutants being downregulated (Fig. S7b), the low level of hydrogen deuteride (HD) formation in samples (Fig. S2) indicates the involvement of Hup in H_2_ recycling, where the reversible component (H/D exchange) is less pronounced. Our results thus highlight a regulatory network between the two metabolic processes in different compartments: the Flv3A‐mediated metabolic processes in vegetative cells and the H_2_ metabolism in heterocysts. It may be that the amount of reducing equivalents in vegetative cells, affected by the activity of Flv3A, has a regulatory role on H_2_ metabolism in heterocysts. However, the nature of the molecular signal that would ultimately regulate gene expression in heterocysts remains unknown. While *in vivo* evidence in *Anabaena* is lacking, reducing equivalents and metabolites may be interchanged between vegetative cells and heterocysts, inducing changes in metabolism and gene expression (Malatinszky *et al*., 2017). A majority of the NADPH needed for the nitrogen metabolism in heterocysts derives from the oxidative pentose phosphate pathway breaking down carbohydrates imported from vegetative cells (Cumino *et al*., 2007), but it is plausible that a more direct exchange of cofactors also occurs, analogously to the malate redox shuttle between the cytosol and chloroplasts in plants and algae. On the other hand, computational modelling suggests that N_2_ uptake rate in heterocysts is limited by the supply of fixed carbon (glutamate) from vegetative cells for incorporation of ammonia (Malatinszky *et al*., 2017). A diminished CO_2_ fixation rate (Fig. 4a) and, possibly, a decrease in GOGAT activity in vegetative ∆*flv3A* cells may therefore induce changes in heterocyst H_2_ metabolism and gene expression. Nevertheless, the molecular mechanism underlying the regulatory network between different cell types needs further elucidation.

Taken together, our results demonstrate that vegetative‐cell‐specific AnaFlv1A and AnaFlv3A are indispensable under harsh fluctuating light conditions regardless of nitrogen or CO_2_ availability, most likely maintaining sufficient oxidation of the photosynthetic electron transport chain and pmf generation for ATP synthesis, and allowing energization of the CCM by catalysing the Mehler‐like reaction as AnaFlv1A/Flv3A hetero‐oligomers. Under LC, AnaFlv3A facilitates moderate O_2_ photoreduction independently of AnaFlv1A in coordination with AnaFlv2 and AnaFlv4. Deletion of AnaFlv3A caused downregulation of the heterocyst‐specific Hup enzyme, resulting in increased light‐induced net H_2_ production. This novel regulatory network between photosynthesis and diazotrophic metabolism might represent an unexploited source of future biotechnological applications.

## Author contributions

YA conceived the study. AS‐S, LN, EW, GT, ME, SK and YA designed the research. AS‐S and LN performed measurements of Chl fluorescence and MIMS. LN performed Dual‐KLAS/NIR, the CCM coefficient assay, and ECS DIRK measurements. AS‐S and EW performed Western blotting and RT‐qPCR. ME performed growth characterization of the mutants and several preliminary experiments. SK measured H_2_ production using the electrode. MH and SK performed deuterium uptake experiments. GT performed RT‐qPCR and part of the Dual‐PAM‐100 experiments, and JW constructed an independent ∆*flv3A* mutant. E‐MA provided resources. AS‐S and LN drafted the manuscript, and all authors revised and approved it. AS‐S and LN contributed equally to this work.

## Supporting information


**Fig. S1** Construction of the *Anabaena* ∆*flv1A*, ∆*flv3A* and ∆*flv1A/3A* mutants.
**Fig. S2** Hydrogen (H_2_), deuterium (D_2_) and hydrogen deuteride (HD) fluxes in *Anabaena* filaments.
**Fig. S3** Generation of proton motive force (pmf) during dark‐to‐light transitions in wild‐type, ∆*flv1A*, and ∆*flv3A* filaments.
**Fig. S4** Growth characterization of *Anabaena* wild‐type, ∆*flv1A* and ∆*flv3A* filaments.
**Fig. S5** Micrographs of diazotrophic *Anabaena* wild‐type, ∆*flv1A*, and ∆*flv3A* filaments.
**Fig. S6** Characterization of *Anabaena* wild‐type, ∆*flv1A* and ∆*flv3A*.
**Fig. S7** H_2_ metabolism in diazotrophic filaments of *Anabaena* wild‐type and flavodiiron protein mutants.
**Fig. S8** DUAL‐KLAS‐NIR kinetics of the primary electron donor of photosystem I (P700), plastocyanin (PC) and ferredoxin (Fd) in *Synechocystis* ∆*flv1* mutant.
**Fig. S9** Differential model blots (DMPs) for deconvolution of PC, P700, and Fd signals with the DUAL‐KLAS‐NIR spectrometer.
**Fig. S10** O_2_ exchange rates of the nondiazotrophic *Synechocystis* ∆*flv1* mutant.
**Fig. S11** Fluorescence induction curves of diazotrophic *Anabaena* ∆*flv1A/3A* cultivated under 0.04% CO_2_ (LC) or 1% CO_2_ (HC) conditions.
**Fig. S12** Effect of plastid terminal oxidase on residual O_2_ photoreduction in diazotrophic *Δflv1A* filaments.
**Fig. S13** Gas exchange analysis of the diazotrophic *Anabaena* filaments.
**Fig. S14** H_2_ and O_2_ fluxes in the *Anabaena* wild‐type and ∆*hupL* filaments.
**Methods S1** Determination of P700 and Fd redox changes from near‐infrared absorbance.
**Methods S2** Determination of the 77 K fluorescence spectra and the transient post‐illumination fluorescence (F_0_ rise).
**Table S1** Oligonucleotide sequences used for quantitative polymerase chain reaction.
Please note: Wiley Blackwell are not responsible for the content or functionality of any Supporting Information supplied by the authors. Any queries (other than missing material) should be directed to the *New Phytologist* Central Office.

## Data Availability

The data that support the findings of this study are available in the Supporting Information which accompanies this article and from the corresponding author upon reasonable request.
