# Supplementary material for: Flv3A facilitates O2 photoreduction and affects H2 photoproduction independently of Flv1A in diazotrophic Anabaena filaments
Source: New Phytol. 2022 Oct 11;237(1):126–39. doi: 10.1111/nph.18506 (PMC10092803; doi:10.1111/nph.18506)
Supplement: Supplementary file 1 — Fig. S1 Construction of the Anabaena ∆flv1A, ∆flv3A and ∆flv1A/3A mutants. Fig. S2 Hydrogen (H2), deuterium (D2) and hydrogen deuteride (HD) fluxes in Anabaena filaments. Fig. S3 Generation of proton motive force (pmf) during dark‐to‐light transitions in wild‐type, ∆flv1A, and ∆flv3A filaments. Fig. S4 Growth characterization of Anabaena wild‐type, ∆flv1A and ∆flv3A filaments. Fig. S5 Micrographs of diazotrophic Anabaena wild‐type, ∆flv1A, and ∆flv3A filaments. Fig. S6 Characterization of Anabaena wild‐type, ∆flv1A and ∆flv3A. Fig. S7 H2 metabolism in diazotrophic filaments of Anabaena wild‐type and flavodiiron protein mutants. Fig. S8 DUAL‐KLAS‐NIR kinetics of the primary electron donor of photosystem I (P700), plastocyanin (PC) and ferredoxin (Fd) in Synechocystis ∆flv1 mutant. Fig. S9 Differential model blots (DMPs) for deconvolution of PC, P700, and Fd signals with the DUAL‐KLAS‐NIR spectrometer. Fig. S10 O2 exchange rates of the nondiazotrophic Synechocystis ∆flv1 mutant. Fig. S11 Fluorescence induction curves of diazotrophic Anabaena ∆flv1A/3A cultivated under 0.04% CO2 (LC) or 1% CO2 (HC) conditions. Fig. S12 Effect of plastid terminal oxidase on residual O2 photoreduction in diazotrophic Δflv1A filaments. Fig. S13 Gas exchange analysis of the diazotrophic Anabaena filaments. Fig. S14 H2 and O2 fluxes in the Anabaena wild‐type and ∆hupL filaments. Methods S1 Determination of P700 and Fd redox changes from near‐infrared absorbance. Methods S2 Determination of the 77 K fluorescence spectra and the transient post‐illumination fluorescence (F0 rise). Table S1 Oligonucleotide sequences used for quantitative polymerase chain reaction. Please note: Wiley Blackwell are not responsible for the content or functionality of any Supporting Information supplied by the authors. Any queries (other than missing material) should be directed to the New Phytologist Central Office. [file NPH-237-126-s001.pdf]

New Phytologist

***New Phytologist* Supporting Information**

Article title: Flv3A facilitates O_2 photoreduction and affects H_2 photoproduction independently of Flv1A in diazotrophic *Anabaena* filaments

Anita Santana-Sánchez**,** Lauri Nikkanen, Elisa Werner, Gábor Tóth, Maria Ermakova, Sergey Kosourov, Julia Walter, Meilin He, Eva-Mari Aro, Yagut Allahverdiyeva

Article acceptance date: 10th September 2022.

The following Supporting Information is available for this article:

Fig. S1 Construction of the *Anabaena* Δf*lv1A*, Δ*flv3A* and Δ*flv1A/3A* mutants.

Fig. S2 H_2, D_2, and HD fluxes in *Anabaena* filaments.

Fig. S3 Generation of *pmf* during dark-to-light transitions in WT, Δ*flv1A*, and Δ*flv3A* filaments.

Fig. S4 Growth characterization of *Anabaena* WT, Δf*lv1A* and Δ*flv3A* filaments.

Fig. S5 Micrographs of diazotrophic *Anabaena* WT, Δf*lv1A*, and Δ*flv3A* filaments.

Fig. S6 Characterization of *Anabaena* WT, Δ*flv1A* and Δ*flv3A.*

Fig. S7 H_2 metabolism in diazotrophic filaments of *Anabaena* WT and FDP mutants.

Fig. S8 DUAL-KLAS-NIR kinetics of P700, PC and Fd in *Synechocystis* Δ*flv1* mutant.

Fig. S9 Differential model blots (DMPs) for deconvolution of PC, P700, and Fd signals with the DUAL-KLAS-NIR spectrometer.

Fig. S10 O_2 exchange rates of the non-diazotrophic *Synechocystis* Δ*flv1* mutant.

Fig. S11 Fluorescence induction curves of diazotrophic *Anabaena* Δ*flv1A/3A* cultivated under LC or HC.

Fig. S12 Effect of PTOX on residual O_2 photoreduction in diazotrophic *Δflv1A* filaments.

Fig. S13 Gas exchange analysis of the diazotrophic *Anabaena* filaments.

Fig. S14 H_2 and O_2 fluxes in the *Anabaena* Δ*hupL* filaments.

Table S1 Oligonucleotide sequences used for qPCR.

Methods S1 Determination of P700 and Fd redox changes from near-infrared absorbance.

Methods S2 Determination of the 77K fluorescence spectra and the transient post-illumination fluorescence (F_0 rise).

Supplementary Figures

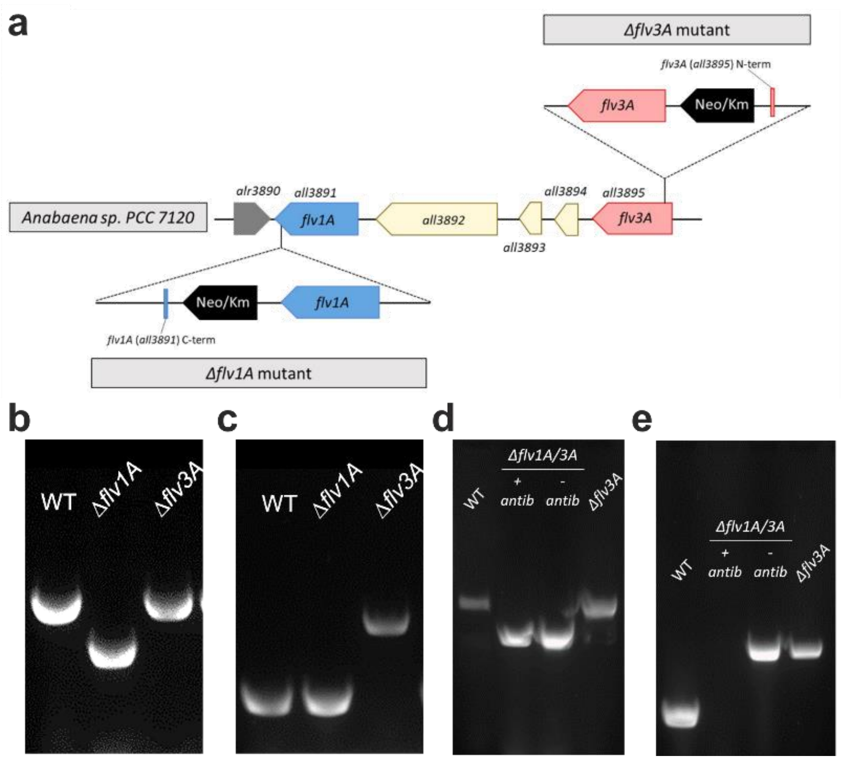

Fig. S1. Construction of the *Anabaena* Δf*lv1A*, Δ*flv3A* and Δ*flv1A/3A* mutants. (a) Genomic structure of the mutants. (b-e) PCR tests of the mutants to confirm full genomic segregation using primers (see Table S1) for *flv1A* (b and d) and *flv3A* (c and e).

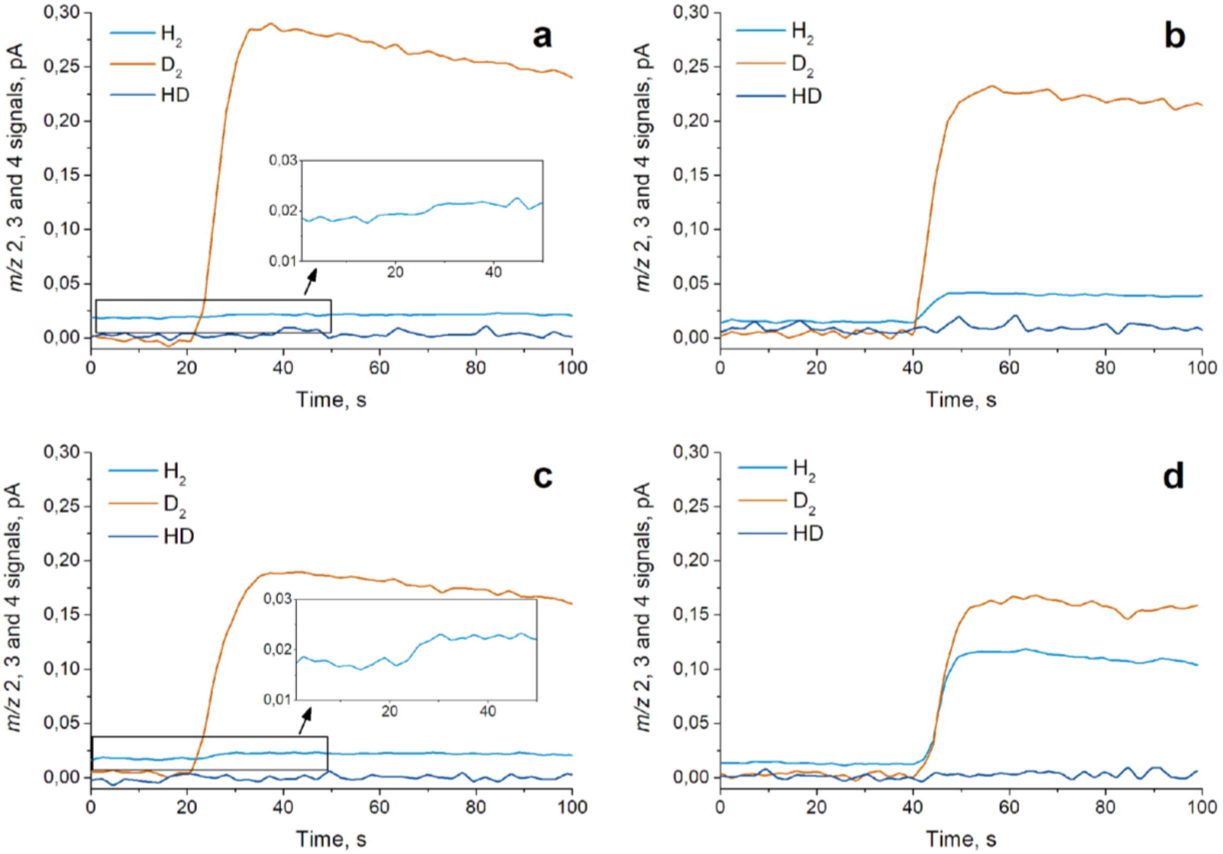

Fig. S2. H_2, D_2, and HD fluxes in *Anabaena* filaments. The response of *m/z* 2 (H_2), 3 (HD), and 4 (D_2) signals on the injection of 250 µL samples (at around 20 and 40 s) into the sealed MIMS chamber, which were taken from the headspace of the vials at 2 h (a, c) and 24 h (b, d) after the introduction of 2% D_2 in the vials. a and b panels demonstrate changes of the signals in the wild-type strain, while c and d show the changes in the Δ*flv1A/3A* mutant. Inserts, representing H_2 signals in the WT (a) and Δ*flv1A/3A* (c) strains, show that the mutant accumulates H_2 in the headspace of the vials already after 2 h. The total H_2 consumption activity in the samples was estimated as a reduction of the D_2 content in the headspace of the vials after 22 h of incubation under 50 µmol photons m^{-2} s^{-1} light at 30 °C. This activity may include the consumption of D_2 by both Hup / Hox hydrogenases. Since the reaction occurs under argon atmosphere, the contribution of nitrogenase in D_2 uptake *via* the H/D exchange reaction is not considered. Each curve represents an average obtained from 6 vials (two biological samples with three technical repeats).

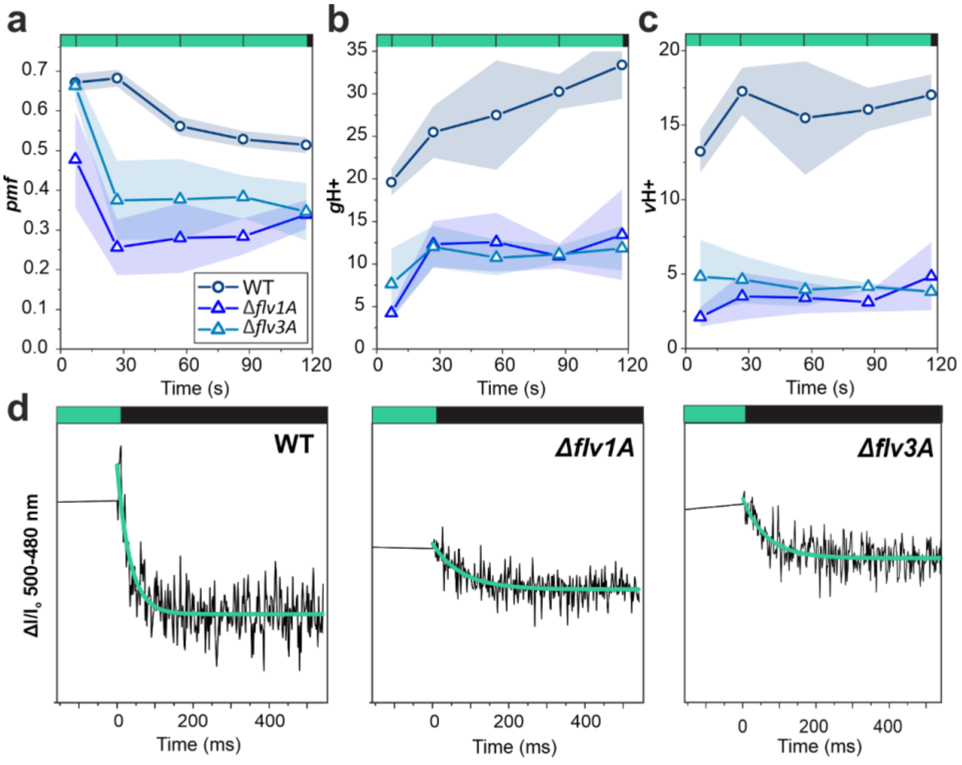

Fig. S3. **Generation of *pmf* during dark-to-light transitions in WT, Δ*flv1A*, and Δ*flv3A* filaments.** Cells were illuminated with 500 µmol photons m^{-2} s^{-1} of green actinic light for 2 min. The proton motive force (*pmf*, a), thylakoid conductivity to protons (*g*H+, b), and proton flux (*v*H+, c) were determined from dark interval relaxations kinetics (DIRK) of the electrochromic shift signal (ECS) as described in the legend for Fig. 5, which represents the second timepoint from these measurements. Shadowing represents SEM. (d) Representative traces of the post-illumination relaxation kinetics of the ECS signal after 27 s of illumination in WT, Δ*flv1A*, and Δ*flv3A* with first order fits used to determine *g*H+ and *v*H+ shown in green.

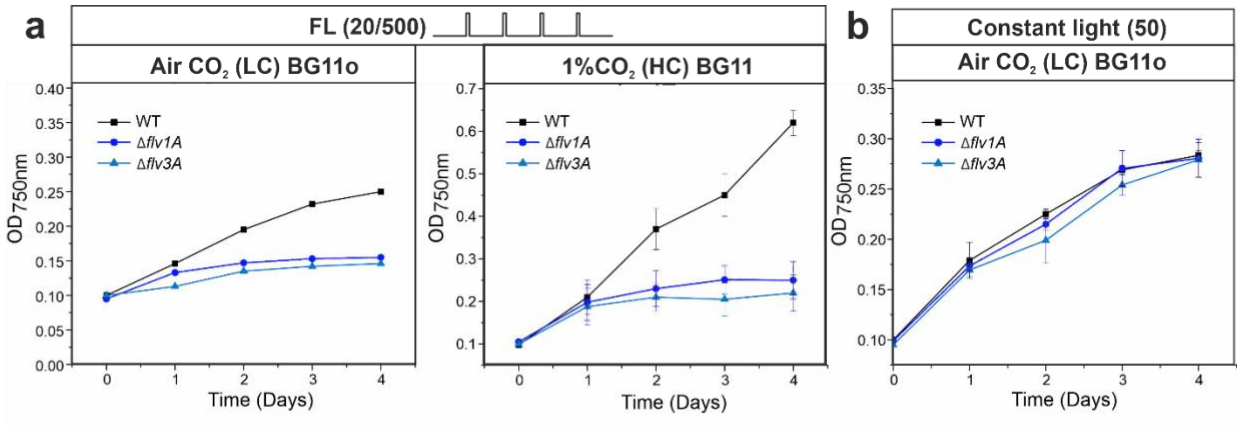

Fig. S4. Growth characterization of *Anabaena* WT, Δ*flv1A* and Δ*flv3A* filaments. (a) Growth curves of filaments grown under severe fluctuating light (FL 20/500) through bubbling with air or 1% CO_2. (b) Diazotrophic growth in BG-11_0 and air bubbling. The values are averages of 3 biological replicates ± SD.

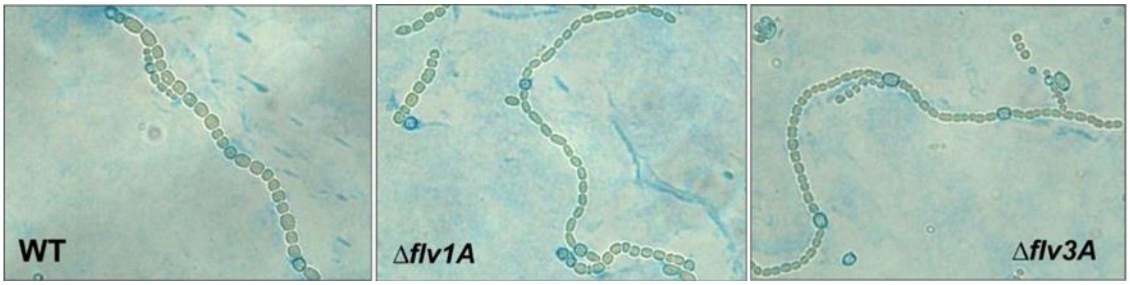

Fig. S5. Micrographs of diazotrophic WT, Δ*flv1A*, and Δ*flv3A* filaments.

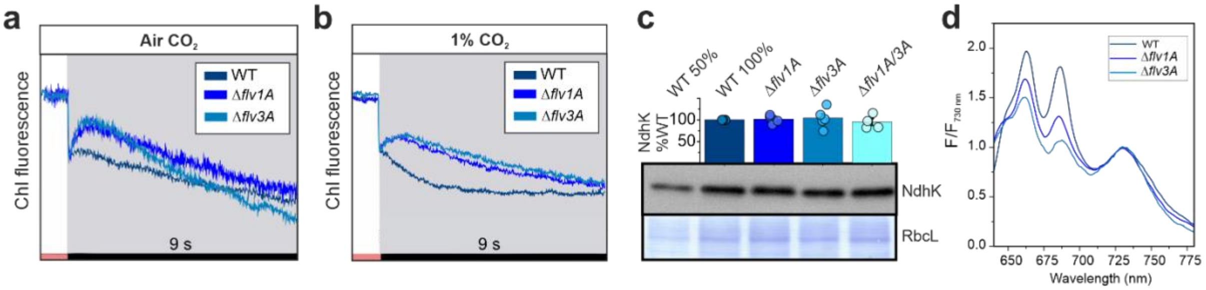

Fig. S6. Characterization of *Anabaena* WT, Δ*flv1A*, and Δ*flv3A* filaments. (a) Transient post-illumination fluorescence rise was monitored after switching off the red actinic light (red bars) for filaments grown in air or (b) 1 % CO_2. Representative traces of 3 biological replicates are shown. (c) Immunodetection of the NdhK subunit of the NDH-1 complex from cells grown in air level CO_2. Coomassie blue -stained band of the large Rubisco subunit is shown as loading control. The column charts represent quantified averages ± SEM from 4-5 biological replicates with all intensities normalised to the WT level, and with individual data points shown by circles. (d) 77K fluorescence emission spectra from dark-adapted WT, *Δflv1A*, and *Δflv3A* filaments. The peaks at 685 nm and 660 nm derive from PSII and phycobilisomes, respectively. Representative spectra from 3 biological replicates are shown. Lower PSII peak relative to PSI reflects a more pronounced state II in the dark.

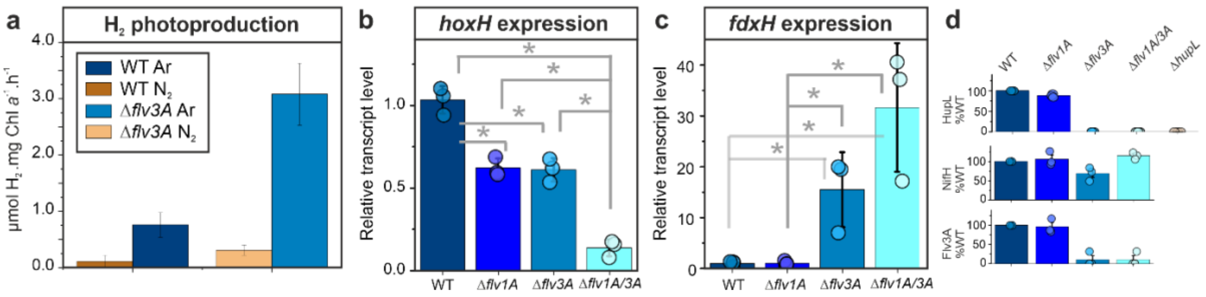

Fig. S7. H_2 metabolism in diazotrophic filaments of *Anabaena* WT and FDP mutants. (a) Specific H_2 photoproduction rates under an Ar or N_2 atmosphere. Rates were normalised to Chl *a* concentration of the sample and calculated using linear regression. Transcript abundance of *hoxH* (b) and *fdxH* (c) relative to WT level. (d) Quantification of Western blot band intensities for HupL, NifH (see Fig. 6c), and Flv3A immunoblots (see Fig. 1d). Values are averages from 3 biological replicates ±SEM. Circles in (b-d) represent individual replicate data. Asterisks in (b-c) indicates significant differences according to Student's t-tests ($P<0.05$).

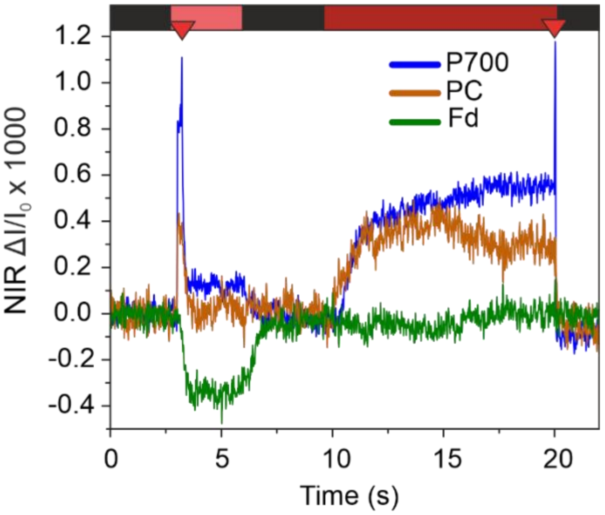

Fig. S8. DUAL-KLAS-NIR kinetics of P700, PC and Fd in *Synechocystis* Δ*flv1* mutant.
The Δ*flv1* mutant was grown in ambient CO_2 level and illuminated with 50 µmol photons $m^{-2}s^{-1}$ for 4 days in BG-11 pH 7.5, after which cells were harvested and Chl *a* concentration adjusted to 10 µg mL^{-1} with fresh BG-11. Maximum amplitudes of PC, P700 and Fd in Δ*flv1* cells were determined with the NIRMAX script of the DUAL-KLAS-NIR software. 3 s illumination of red AL (200 µmol photons $m^{-2}s^{-1}$), with a saturating pulse (depicted by a red triangle) after 200 ms of illumination to fully reduce the Fd pool. After 4 s of darkness thereafter, cells were illuminated with far-red light for 10 s with a saturating pulse in the end to fully oxidize P700.

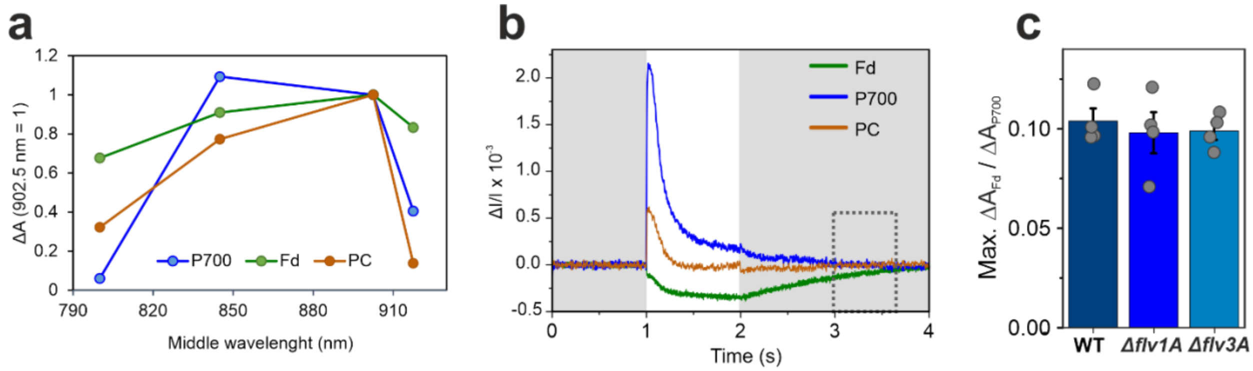

Fig. S9. Differential model blots (DMPs) for deconvolution of PC, P700, and Fd signals with the DUAL-KLAS/NIR spectrometer. (a) DMPs determined from WT (P700 and PC) and Δ*flv1A*/Δ*flv3A* (Fd) *Anabaena* cells according to protocols described by Theune et al. (2021), with the exception that the Δ*flv1A*/Δ*flv3A* mutant was used instead of anoxic conditions to inhibit the Mehler-like reaction and thus delay reoxidation of Fd sufficiently to determine the Fd DMP. The values are normalized to the ΔA value at the middle wavelength at 902.5 nm. (b) Deconvoluted traces from the determination of the Fd DMP. Δ*flv1A*/Δ*flv3A* cells were illuminated for 1 s at 1350 µmol photons m^{-2} s^{-1} to fully reduce the Fd pool. The illumination was repeated 10 times with a 30 s interval in-between measurements, and averaged traces from the 10 measurements are shown. The dashed rectangle indicates the time window where only Fd redox changes occur and which was used to determine the Fd DMP. (c) Ratio of maximal NIR absorbance change attributable to Fd / P700, as determined by a modified NIRMAX script for the Dual-KLAS/NIR and the DMPs in (a). Values are averages from 4 biological replicates ± SEM, with individual datapoints shown as circles.

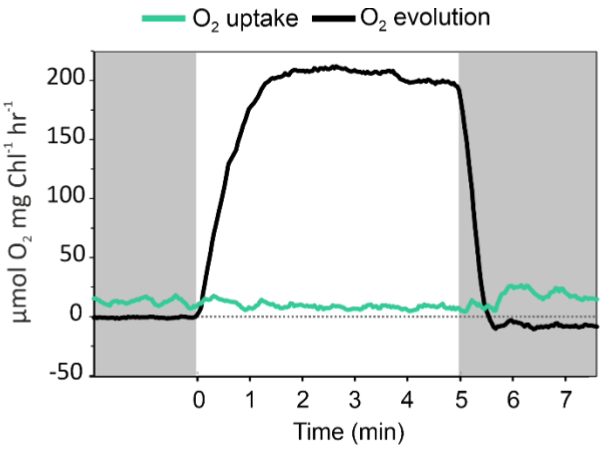

Fig. S10. O_2 exchange rates of the *Synechocystis* Δ*flv1* mutant. Experimental cultures were grown in BG-11 medium in air level of CO_2 and illuminated with 50 µmol photons m^{-2} sec^{-1} light for 4 days. After this, the cells were harvested and Chl *a* concentration adjusted to 10 µg mL^{-1} with fresh BG-11. Cells were dark-adapted for 15 min, and gas exchange was monitored by MIMS over a 5-min illumination period with 500 µmol photons m^{-1} s^{-2} of white actinic light. Before the measurements, samples were supplemented with 1.5 mM $NaHCO_3$.

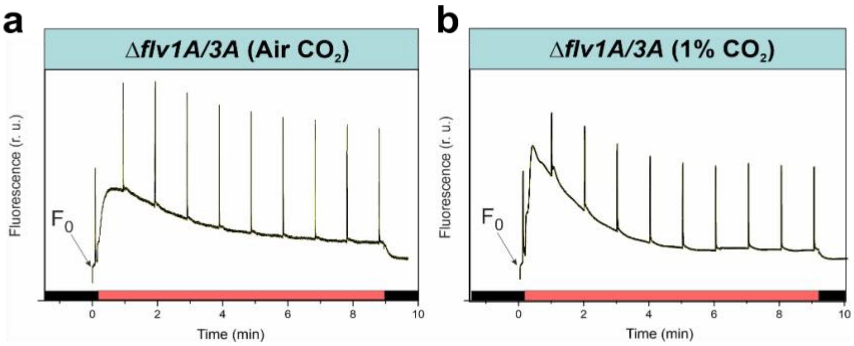

Fig. S11. Fluorescence induction curves of diazotrophic *Anabaena* Δ*flv1A*/*flv3A* cultivated under LC or HC. (a, b) The kinetics are representative of three biological replicates. Cells were dark acclimated for 10 min before the measurement.

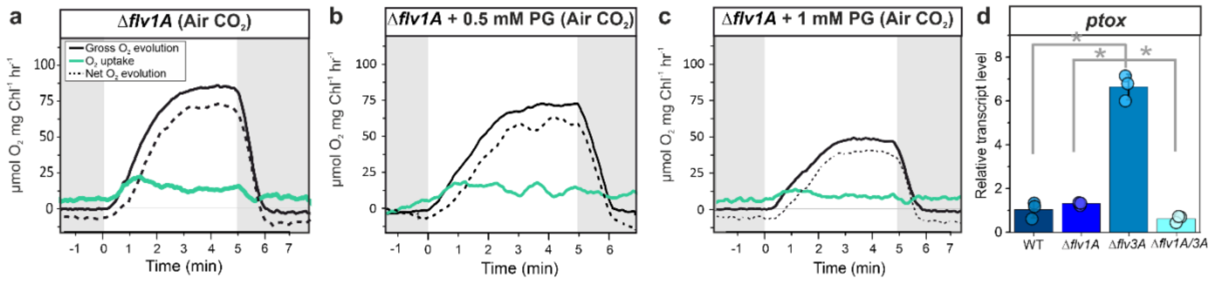

Fig. S12. Effect of PTOX on residual O_2 photoreduction in diazotrophic *Δflv1A* filaments. O_2 exchange was monitored by MIMS without (a) and in presence of 0.5 mM (b) or 1 mM of n-propyl gallate (c). MIMS measurements were carried out as described in the legend for Fig. 3. (d) Transcript levels of *ptox* in diazotrophic WT, *Δflv1A, Δflv3A and Δflv1A/3A* filaments grown under air level CO_2 as determined by RT-qPCR. The values are averages of 3 biological replicates ± SD with individual replicate data shown as circles and significant differences according to Student's t-tests (P<0.05) are indicated by *.

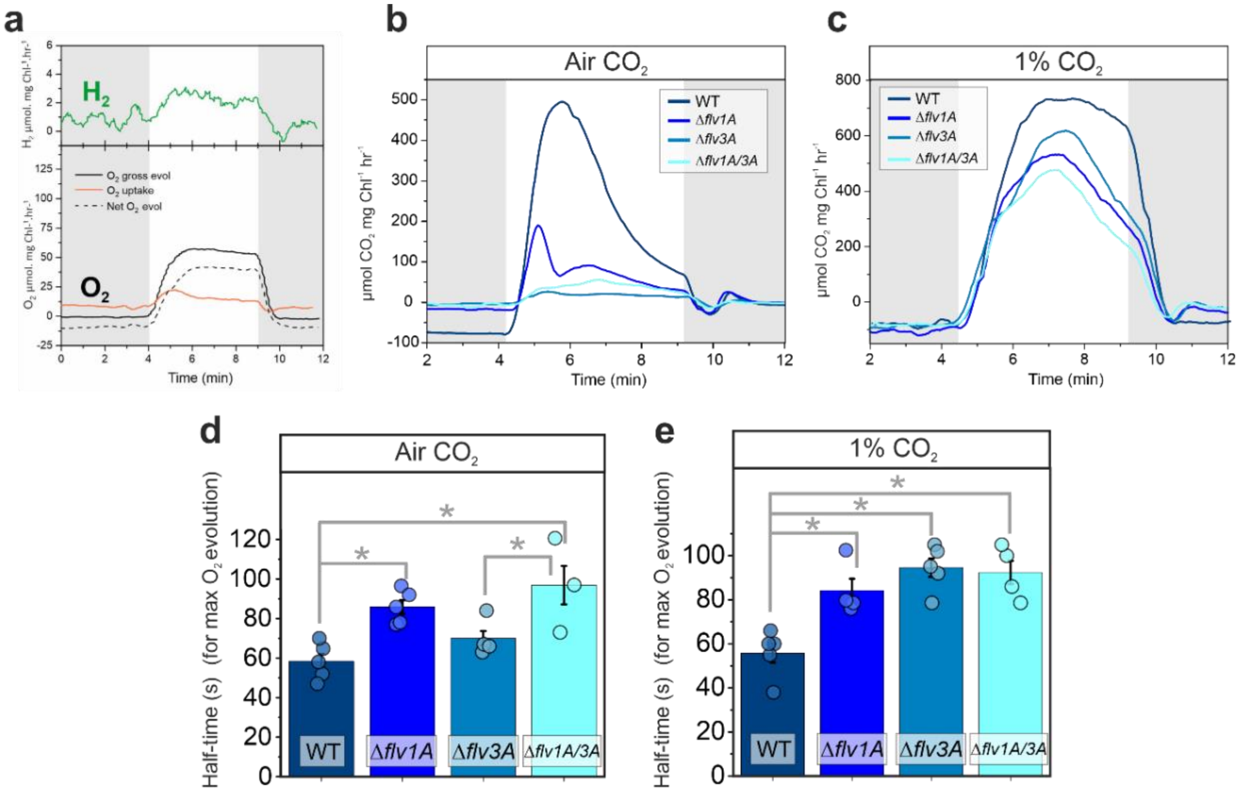

Fig. S13. Gas exchange analysis of diazotrophic *Anabaena* filaments. (a) O_2 and H_2 exchange rates of a second independent mutant colony Δ*flv3A*_C2 grown under LC. (b, c) CO_2 uptake rates of WT, Δ*flv1A*, and Δ*flv3A* filaments grown under LC (b) or HC (c). Changes in CO_2 concentration were recorded simultaneously with the measurements in Fig 3. Gas exchange rates are presented as μmol mg Chl a^{-1} h^{-1}. Representative traces of 4-5 biological replicates are shown. (d, e) Half-times of the induction of maximal O_2 evolution under LC (air) conditions (d) and HC (1% CO_2) in (e). Data in (d-e) are from measurements for Fig. 3. Averages from 3-5 (d) and 4-5 (e) biological replicates ± SE are shown as columns and individual data points as circles. Statistically significant differences according to Student's t-tests (P<0.05) are indicated by *.

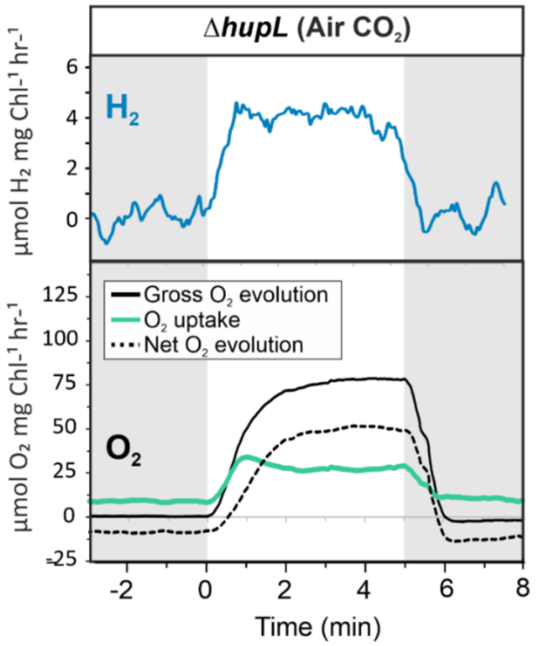

Fig. S14. H_2 and O_2 fluxes in the *Anabaena* Δ*hupL* mutant. Experimental conditions were as described in the legend for Figure 3.

Supplementary Tables

Table S1 Oligonucleotide sequences used for qPCR.

**Gene name**	**Forward primer (5' →3')**	**Reverse primer (3' →5')**
flv1A (all3891)	tgcagaaacgactagatgtca	aaatccgggttttctagcac
flv3A (all3895)	ggtactacatataactcatttctcatacgc	tgactaagccactgtggtctgg
flv2 (all4444)	actctccaaagcaccacaag	taaacaaaccatccggccaa
flv4 (all4446)	ctgctattcgctgtttggat	ttcactaagccgctatggtc
hoxH (alr0766)	gggacaaatcctccaatccc	tttgctcctcccaacacttc
hupL (all0687)	agtagccgcttctacgatga	acccaaccacacaggttcta
ptox (all2096)	cctatcctcgcttttatgtactagaaacc	cacctcccaaagactccataatcaac
fdxH (all1430)	taccaagttagattgatc	aagtaacacaaagtagag
nifH (all1455)	ttctacaaacccctcacagc	gatttaccgataccgccttt
rnpB	ggactaggggttggggact	acgagggcgattatctatctg

Supplementary Materials and Methods

Methods S1. Determination of P700 and Fd redox changes from near-infrared absorbance. Redox change kinetics of P700 and Fd were deconvoluted from the four difference signals using differential model plots (model spectra Fig. S9) that were measured for *Anabaena* using protocols described earlier (Theune et al., 2021) with the modification for the Fd model spectrum, we used the Δ*flv1A*/Δ*flv3A* mutant instead of anoxic conditions to impair the Mehler-like reaction (see Fig.3) and to sufficiently slow down the re-oxidation Fd. For P700 and plastocyanin (PC) model spectra, we used WT *Anabaena* filaments. Due to low signal quality, the PC traces were omitted from Fig. 2. As noted for *Synechocystis* earlier (Theune et al., 2021), it is likely that the redox kinetics of P700 and PC in *Anabaena* may be closely related, thus making it difficult to extract a PC signal of large magnitude.

Methods S2. Determination of the 77K fluorescence spectra and the transient post-illumination fluorescence (F_0 rise). The fluorescence emission spectra at 77K were measured using QE Pro-FL spectrofluorometer (Ocean Optics). Samples of diazotrophic filaments were adjusted to a Chl a concentration of 5 µg mL^{-1}, dark-adapted for 15 min and rapidly frozen in liquid nitrogen. Excitation wavelength was 580 nm and fluorescence spectra were normalised to the PSI peak at 730 nm.

The F_0 rise was monitored for 9 s after the termination of actinic light from measurements shown in Fig. 1a-b (9 min illumination with red actinic light at 50 µmol photons m^{-2} s^{-1}). The traces were normalised to F_s to facilitate a comparison of the kinetics.

Reference:

Theune ML, Hildebrandt S, Steffen-Heins A, Bilger W, Gutekunst K, Appel J. 2021. In-vivo quantification of electron flow through photosystem I–Cyclic electron transport makes up about 35% in a cyanobacterium. *Biochimica et Biophysica Acta - Bioenergetics* **1862:** 148353.
